# Molecular Characterization and Event-Specific Real-Time PCR Detection of Two Dissimilar Groups of Genetically Modified Petunia (*Petunia* x *hybrida*) Sold on the Market

**DOI:** 10.3389/fpls.2020.01047

**Published:** 2020-07-14

**Authors:** Marleen M. Voorhuijzen, Theo W. Prins, Anke Belter, Joachim Bendiek, Claudia Brünen-Nieweler, Jeroen P. van Dijk, Ottmar Goerlich, Esther J. Kok, Benjamin Pickel, Ingrid M. J. Scholtens, Andrea Stolz, Lutz Grohmann

**Affiliations:** ^1^ Wageningen Food Safety Research (WFSR), Wageningen University & Research, Wageningen, Netherlands; ^2^ Saxony-Anhalt Environmental Protection Agency (EPA), Halle (Saale), Germany; ^3^ Federal Ministry of Food and Agriculture, Berlin, Germany; ^4^ Chemical and Veterinary Analytical Institute Muensterland-Emscher-Lippe, Muenster, Germany; ^5^ Bavarian Health and Food Safety Authority, Oberschleißheim, Germany; ^6^ Agricultural Analytic and Research Institute, Speyer, Germany; ^7^ Federal Office of Consumer Protection and Food Safety, Berlin, Germany

**Keywords:** petunia, genetically modified (GM), MinION sequencing, amplification of linearly-enriched fragments, event-specific detection, real-time PCR, unauthorized GMO

## Abstract

Petunia plants with unusual orange flowers were noticed on the European market and confirmed to be genetically modified (GM) by the Finnish authorities in spring 2017. Later in 2017, inspections and controls performed by several official laboratories of national competent authorities in the European Union detected several GM petunia varieties with orange flowers, but also another group of unusually colored flowers. In the latter group, a so far undetected gene coding for a flavonoid 3’5’ hydroxylase (F3’5’H) responsible for the purple color was identified by German and Dutch authorities, suggesting that the petunias found on the markets contain different genetic constructs. Here, a strategy is described for the identification of GM petunia varieties. It is based on an initial GMO screening for known elements using (real-time) PCR and subsequent identification of the insertion sites by a gene walking-like approach called ALF (amplification of linearly-enriched fragments) in combination with Sanger and MinION sequencing. The results indicate that the positively identified GM petunias can be traced back to two dissimilar GM events used for breeding of the different varieties. The test results also confirm that the transgenic petunia event RL01-17 used in the first German field trial in 1991 is not the origin of the GM petunias sold on the market. On basis of the obtained sequence data, event-specific real-time PCR confirmatory methods were developed and validated. These methods are applicable for the rapid detection and identification of GM petunias in routine analysis. In addition, a decision support system was developed for revealing the most likely origin of the GM petunia.

## Introduction

In spring 2017 Finnish authorities first reported that ornamental petunias (*Petunia* x *hybrida* or *P. hybrida*) placed on the market were genetically modified (GM)^1^. These plants have an unusual orange flower color that could not have been generated by conventional breeding.

Molecular analyses showed that a specific GM construct in orange petunias is responsible for the flower color (Bashandy and Teeri, 2017). The GM construct was linked to a genetically engineered plasmid that had, three decades earlier, been used to develop petunias with a modified flower color as a marker trait to investigate plant transposons (Meyer et al., 1987). At that time research at the Max Planck Institute for Plant Breeding Research (MPI) in Cologne included the first open-field trials of GM plants in Germany in 1991^2^.

The GM petunia sale as gardening plants in Finland was notified to the European Commission, who immediately informed all member states that no application for the import, cultivation, or marketing of GM petunia was ever submitted in the European Union (EU). Authorization of cultivation of a GM petunia would have to take place under the Directive 2001/18/EC and a report on risk assessment and a monitoring once placed on the market would be required (European Commission, 2001). In subsequent inspections and controls, various petunia varieties having orange, but also otherwise unusual colored flowers were tested GM-positive by the official laboratories of the national competent authorities in the EU. It became apparent, that in several Member States the domestic breeding companies were affected and had unintentionally traded these GM petunia events in their downstream distribution and marketing chains for many years^3^. The same situation was promptly revealed in the United States after it was officially announced, and in October 2017 it was reported that at least 124 varieties of GM petunias had been unintentionally imported and distributed interstate without proper US authorization^4^.

With regard to the aspect of risk to plants, animals, or human health, Dutch and German independent scientific advisory committees were asked to provide advice on the possible impact of the unauthorized release of these GM petunias on the environment^5^ ^,^ ^6^. According to the opinions of these committees the GM garden petunias with altered flower color pose a negligible risk to humans and the environment. Nevertheless, due to the lack of any authorization under applicable law in the EU, the US or elsewhere, all petunia plants and varieties had to be completely removed from the market and as well from further trade and were, according to breeder’s information, destroyed.

Parts of the civil society but also some breeders speculated that the finding of GM petunias sold on the market originated from the deliberate release of GM petunia plants during the first field trials in Germany in 1990/91. In this field trial, transgenic petunia plants with a maize A1 gene (dfr-MAIZE) coding for a dihydroflavonol 4-reductase (DFR) were used (Meyer et al., 1987). The dfr-MAIZE converts endogenously formed dihydrokaempferol to leukopelargonidin, resulting in the production of the pigment pelargonin and therefore a salmon red flower color. About 30,000 GM petunias of the so-called event RL01-17 were planted in this trial^2^. However, because of exposition to extreme sunlight, the experiment failed its goal to detect the action of plant transposons. This finding later on led to evidence that endogenous and environmental epigenetic effects can influence gene expression and, in this specific case, intensive sunlight can change the flower color (Linn et al., 1990; Meyer et al., 1992). The reports on the development of new flower colors stimulated breeders to use suitable GM events with an inserted maize dfr gene with the objective to produce attractive and stably orange-flowering garden petunia varieties for commercialization (Oud et al., 1995).

Following the Finnish findings, many different petunia varieties sold under imaginative trade names on the market were analyzed in Germany and the Netherlands. Using real-time PCR methods targeting the 35S promoter (P-35S), the nos terminator (T-nos), the nopalinphosphotransferase II gene (nptII), or a construct (P-35S/nptII), in the suspicious GM petunias these sequences were present. The methods were thus found suitable for detection of the genetic modifications³. During the analyses it became evident that not all elements were detected in a subset of samples taken from pink flowering petunias. Initial gene walking experiments were conducted and a gene coding for a different color modifying gene (F3’5’H) was identified in these petunias, suggesting the existence of another group of GM petunias on the market.

Recent in-depth investigation of the presence and nature of the dfr-MAIZE gene construct and its impact on the flavonoid metabolism has shed additional light on the possible origin of the GM petunias (Haselmair-Gosch et al., 2018). However, these authors did not exactly identify the GM event(s) and their origin. In another recently published study, the MinION sequencing technology was applied to investigate the nature of the GM construct and the insertion site in 23 different petunia varieties (Fraiture et al., 2019). The study suggests that the orange flowering petunia varieties have an identical insertion site and a similar origin of GM event transformation. However, the origin of the parental events remained undiscovered. In addition, a “petition for the determination of non-regulated status” for 23 lines containing the dfr-MAIZE was recently submitted to the US competent authority (USDA-APHIS, 2019). According to the petition, the progenitors of all lines are different events developed by the MPI (Linn et al., 1990; Meyer et al., 1992; Oud et al., 1995). Potential progenitors are MPI-15 (235/1-15 or RP235-15), MPI-17 (235/1-17 or RL01-17), and RL01-24 (or 235/1-24). However, no detailed GM event characterization is provided, and it remains unclear which GM event carrying the dfr-MAIZE gene was illegally sold on the market.

Without knowledge of the insertion site of a transgene construct in the plant genome, a GM event is not fully characterized. Event-specific methods are indispensable for the identification of a GMO and validated event-specific methods can be an efficient resource for the market control of petunia plants.

In this paper, we describe the results of comprehensive element-specific PCR investigations of GM petunia varieties detected during the market control in 2017. The results show that these varieties contain deviating GM constructs and fall in two dissimilar groups named G1 with orange and G2 with pink flowers. In G2 petunias additional GM elements and a different flower color modifying gene were detected.

A representative of each of the two groups was selected for further investigation of the insertion sites using an adapted gene walking approach called ALF (amplification of linearly-enriched fragments) in combination with Sanger sequencing and the MinION sequencing approach, in order to obtain longer sequencing reads (Košir et al., 2017). On basis of the retrieved sequence data event-specific real-time PCR methods were developed and subsequently validated. The results show that event RL01-17 used in the field trial in 1991 is not the origin of the orange flowering petunias illegally sold on the market.

## Materials and Methods

### Plant Materials and DNAs

**Figure 1:**
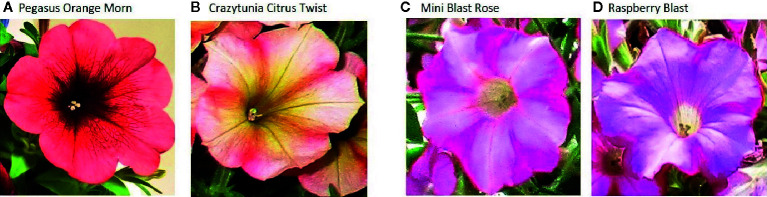
Photographs of individual petunia flowers representing G1 varieties **(A, B)** and G2 varieties **(C, D)**.

Petunia varieties African Sunset, Viva Orange Vein, Viva Fire and Go!Tunia Orange (representing G1), and wild type petunias without cultivar name were provided by the Human Environment and Transport Inspectorate (ILT), the Netherlands. Samples of most of the analyzed petunia varieties (e.g. Pegasus Orange Morn and Crazytunia Citrus Twist, see **Figures 1A, B**) were taken from the German market during the official controls and inspections by the competent authorities (see **Table 1**). Variety names often represent imaginative trade names given by the vendor and may not represent different breeding lines.

**Table 1:** Target-specific PCR test results in comparative analyses of genomic DNAs extracted from different petunia varieties.

Laboratory	Trade nameof petunia variety	Flower color	Target-specific PCR test result (Cq value)^6^					
			actin	P-35S	T-nos	P-nos/nptII	Pxh G1	Pxh G2
WFSR^1^	African Sunset	Orange	24.6	23.6	n.d.	+	23.0	–
LAU^2^	Pegasus Orange Morn	Orange	29.2	+	n.d.	25.9	28.5	–
	Table Orange	Orange	27.3	+	n.d.	26.3	28.7	–
	Landgard	Orange red	27.2	+	n.d.	n.d.	27.9	–
	Potunia Plus Papaya	Orange	26.8	+	n.d.	25.2	27.8	–
	Peppy Red	Red white striped	26.9	+	n.d.	25.4	27.9	–
	Crazytunia Citrus Twist	Yellow red stripes	25.9	+	n.d.	24.3	27.1	–
	Blast Rose	Orange	26.9	+	n.d.	n.d.	27.4	–
	RL01-17	Orange	30.1	+	n.d.	n.d.	–	–
	RL01-24	Orange	25.2	+	n.d.	n.d.	–	–
	Raspberry Blast	Pink (with fading)	26.9	+	+	26.0	–	27.1
	Mini Blast Rose	Pink white striped	25.6	+	+	26.2	–	27.0
LGL^3^	Bonnie Orange	Orange-apricot	22.4	24.9	–	24.4	24.3	–
	Cascadias Red Lips	Red	22.5	24.0	–	23.2	23.2	–
	Potunia Plus Red 2016	Red	23.1	25.4	–	24.5	24.8	–
	Classic Red	Red	22.9	–	–	–	–	–
	Orange yellow center 749 (07336)	Orange	23.2	25.4	–	24.9	25.0	–
	Sentunia (2.0) Gshell Orange	Orange	22.8	24.9	–	24.4	24.6	–
	Charms Flame	Yellow-red	23.3	25.8	–	24.4	25.2	–
	Sentunia 2.0 Rose Coral	Orange apricot	22.2	24.9	–	24.4	24.3	–
	Bingo Coral Blast	Pink/red with white	22.9	25.4	–	24.6	24.8	–
	Crazytunia Citrus Twist	Red	22.8	25.5	–	24.8	24.7	–
	Raspberry Blast (DNA from LAU)	Pink	n.d.	n.d.	n.d.	n.d.	n.d.	28.9
CVUA MEL^4^	Pegasus Orange Morn	Orange	25.7	27.7	n.d.	26.7	26.1	–
	Table Orange	Orange	25.2	27.5	n.d.	25.8	26.2	–
	Pegasus Purple	Purple	26.6	–	n.d.	–	–	–
	Special Mango	Yellow	26.3	–	n.d.	–	–	–
	Pegasus Table Red Star	White-red stripes	25.6	26.7	n.d.	26.2	25.1	–
LUFA^5^	Mini Blast Rose	Pink	22.8	+	+	+	–	26.4
	Supertunia Flamingo	Pink	22.3	+	+	+	–	25.5
	Raspberry Blast	Pink	22.4	+	+	+	–	25.8
	Johnny Flame	Red/salmon	22.7	–	–	–	–	–
	Pegasus Orange	Orange	22.5	+	–	+	23.5	–

Pxh G1, Petunia x hybrida group 1 event-specific test; Pxh G2, Petunia x hybrida group 2 event-specific test; n.d., not determined; +, detected in end-point PCR test; -, not detected in PCR test; ^1^ WFSR, Wageningen Food Safety Research; ^2^LAU, Landesamt für Umweltschutz Sachsen-Anhalt Environmental Protection Agency of Saxony-Anhalt; ^3^LGL, Bayerisches Landesamt für Gesundheit und Lebensmittelsicherheit Bavarian Health and Food Safety Authority; ^4^CVUA MEL, Chemisches und Veterinäruntersuchungsamt Münsterland-Emscher-Lippe Chemical and Veterinary Analytical Institute Münsterland ‐ Emscher ‐ Lippe; ^5^LUFA, Landwirtschaftliche Untersuchungs- und Forschungsanstalt Speyer Agricultural Analytic and Research Institute Speyer; ^6^Cq, quantitation cycle.

Petunia varieties Raspberry Blast and Mini Blast Rose (representing G2) were provided by the Environmental Protection Agency of Saxony-Anhalt, Germany (**Figures 1C, D**). Seeds of petunia events RL01-17 and RL01-24, DNA of plasmids p35SA1, pGS/MDF-17 (contains a 7 kb *Hin*dIII A1-fragment of event RL01-17), and pGS/MDF-24 (contains a 7 kb *Hin*dIII A1-fragment of event RL01-24) originating from the year 1990 and the corresponding complete sequence data of the cloned *Hin*dIII fragments were kindly provided by Prof. Peter Meyer (University of Leeds, Faculty of Biological Sciences).

All GM petunia plants and materials were analyzed in laboratory facilities approved for genetic engineering work.

### DNA Extraction

DNA was isolated from fresh leave material in duplicate using a CTAB extraction followed by DNA purification *via* Qiagen DNeasy plant mini kit (Qiagen) (Scholtens et al., 2013). Approximately one square centimeter plant material was cut from each leaf and transferred into a 2 ml reaction tube. The leaf material was quickly frozen in liquid nitrogen, and the leaf material was ground with a pellet pestle. Cell lysis was performed by adding 150 µl MQ water and 350 µl CTAB-extraction buffer (20 g/l CTAB, 1.4 M NaCl, 0.1 M Tris, and 20 mM Na_2_EDTA, pH 8.0). After vigorously shaking, 5 µl RNase A (Qiagen, 100 mg/ml) was added, the solution was mixed and incubated for 15 min at 65°C in a heated shaker followed by addition of 20 µl proteinase K solution (Life Technologies, 20 mg/ml), vigorously mixing and shaking and further incubation for 1 h at 65°C. Subsequently, the manufacturer’s protocol (Qiagen, DNeasy Plant Handbook March 2018) was followed starting from step 9 with the adjustment of adding 200 µl buffer P3. Quantity and purity of the isolated DNA was determined using a Nanodrop (Nanodrop 1000 instrument, Thermo Fisher Scientific) and evaluated using the A260/A280 and A260/A230 ratios.

### Real-Time PCR

Real-time PCR analyses were performed using screening methods described previously (Scholtens et al., 2013; Scholtens et al., 2017) and targeting the P-35S and T-nos (Kuribara et al., 2002), nptII (Scholtens et al., 2013), T-ocs, T-35S, and P-nos (Debode et al., 2013) elements or the P-nos/nptII construct (ISO/TS 21569-4, 2016). New combinations of downstream and upstream primers of different elements were used to amplify larger insert segments present in GM petunias (Prins et al., 2016).

To select primers and probes for G1 and G2 event-specific real-time PCR methods, the AlleleID tool (PREMIER Biosoft, vs 7.84) was used with default settings for an annealing temperature of 60°C. TaqMan probes were labeled with 6-carboxyfluorescein (FAM) at the 5′ end and Black Hole 1 quencher dye (BHQ1) at the 3′ end (**Table 2**). Real-time PCR was performed in 96-well microtiter plates in a reaction volume of 25 μl, containing 1× universal mastermix (DMMLD2D600, Diagenode, Liège, Belgium), 400 nM of each primer, 200 nM probe, and 50 ng DNA (except for real-time PCR performance). Real-time PCR was performed with the CFX96 real-time PCR detection system (Bio-Rad) with an initial UDG decontamination step at 50°C for 2 min. After an initial denaturation at 95°C for 10 min, 45 cycles were performed (denaturation at 95°C for 15 s and annealing and extension at 60°C for 1 min). All DNA samples were analyzed in duplicate.

**Table 2:** Primer and probe sequences for the event-specific PCR methods.

Target	Name	Sequence 5’-3’	Size(bp)	AmpliconLength (bp)
G1 petunia	Pxh G1-F	GCCACTAATTGGTTATGC	18	93
	Pxh G1-R	GGATGGCATGACAGTAA	17	
	Pxh G1-P	FAM-CAGTGCTGCCATAACCATGAGTG-BHQ1	23	
G2 petunia	Pxh G2-F	GCCAGTGCTCAAACTTAA	18	100
	Pxh G2-R	CTGGATCGGCAATTCAAA	18	
	Pxh G2-P	FAM-CACCTTGGAGGATGACCGCC-BHQ1	20	

### Amplification of Linearly-Enriched Fragments

Amplification of linearly-enriched fragments was performed according to Košir et al. (2017) in separate duplicate reactions for P-35S up- and downstream, T-ocs up- and downstream for the selected G1 petunia variety African Sunset, and P-35S, T-nos, and P-nos up- and downstream for the selected G2 petunia variety Raspberry Blast. Excess of primers and primer-dimers was removed using the QiaQuick PCR purification kit (Qiagen). Streptavidin coated magnetic beads (Dynabeads MyOne Streptavidin C1, Invitrogen) were used to separate the biotinylated fragments from the genomic background. Purified, enriched fragments were poly C-tailed to add the second primer site and amplified in duplicate per enrichment reaction by semi-nested PCR. Subsequently, per enrichment reaction the two duplicate semi-nested PCR reactions, the so-called ALF fragments, were pooled and purified using the QiaQuick PCR purification kit (Qiagen).

### Sanger Sequencing and Data Analysis

Amplicons generated by combining upstream and downstream primers in a conventional PCR and purified ALF fragments were Sanger sequenced by an external service laboratory (Macrogen, Netherlands) using undiluted and 10 times diluted DNA solutions. For uni-directional sequencing 25 pmol of the specific primer was used per reaction. Sequences were analyzed using Geneious (Geneious Biomatters, New Zealand, version R11) and BLAST (Altschul et al., 1997) against GenBank or against specific sequences.

For confirmation of border sequences, primers were designed using the AlleleID tool (Premier Biosoft, vs 7.84). Primers were located approximately 250 nucleotides (nt) upstream and downstream of the expected transition site. For G1 petunia primers S1G1LB-F (GCGGTAAGATCCTTGAGA) and S1G1LB-R (CCGACCAGACATTGCT) were selected to generate an amplicon of 424 bp. For G2 petunia primers S5G2RB-F1 (CAAGATTGTGGTGCTTCA) and S5G2RB-R1 (AAGATATGCGGGTAGAGG) were selected to generate an amplicon of 322 bp.

### MinION Sequencing

Selected ALF fragments (**Table 3**) were taken for further sequence analysis using a MinION sequencer (Oxford Nanopore Technology, ONT). MinION libraries were prepared with 32 or 42 µl (**Table 3**) of purified semi-nested PCR products using the SQK-LSK108 Nanopore Sequencing Kit (ONT) according to the manufacturer’s protocol. Upon arrival and immediately before running flow cells with version R9.4/FLO-MIN106 were quality checked to ensure the presence of at least 800 active biological nanopores using the MinKNOW software (version 1.7.3) as recommended by the manufacturer. ONTs Flow Cell Wash Kit was used to wash the flow cells in between the runs according to the manual. Flow cells were run until between 40,000 and 54,000 reads were generated.

**Table 3:** MinION sequencing data details.

Petunia group	ALF direction	input vol. (µl) library prep	FlowCell	Run	No. of fast5 reads	No. of fastQ sequences	No. of primer selected sequences	No. of sequences matching CaF	No. of clusters > 5 reads per cluster
G1	P-35S up	42	A	1	40,000	14,431	2,247	1,411	13
G1	T-ocs down	42	A	2	47,000	18,696	7,256	0	170
G2	P-nos up	32	A	3	40,000	16,090	172	0	5
G2	P-nos down	32	B	1	40,000	15,195	4,457	3,220	20
G2	T-nos down	32	B	2	40,000	19,624	7,147	0	207
G2	P-35S down	42	B	3	54,000	21,690	6,641	0	151

CaF, known GM plant construct and flanking sequences.

### MinION Data Analysis Pipeline

A data analysis pipeline was used for selection of the most useful reads, i.e. those containing new sequence information (Košir et al., 2017). Individual fast5 reads were quality checked, size selected, primer, and adapter trimmed and finally converted into pre-processed fastQ reads using PRINSEQ software. Briefly, after some QC steps, including primer selection to ensure a relation to the actual enrichment, all reads were examined twice with BLAST, consecutively. First, to remove the reads with a known event sequence and second, to remove all reads fully covered by known GM element sequences. The remaining reads potentially contained useful novel sequence information and were used for consensus building. The consensus sequences underwent a third BLAST analysis, of which the results were presented to the user for interpretation.

### Specificity and Sensitivity Tests of the Event-Specific Methods

G1 and G2 event-specific real-time PCR methods were tested for specificity using different petunia DNA samples (**Table 1**). Real-time PCR efficiency was evaluated in duplicate for G1 (Go!Tunia Orange and Viva Fire) and G2 (Raspberry Blast and Mini Blast Rose) plants in DNA dilution series containing 150, 17.7, 2.08, 0.24, 0.029, 0.014, 0.007, and 0.001 ng per reaction. Based on a haploid petunia genome weight (1C) of 1.43 pg^7^, the estimated copy numbers in the calibration curve were 105634, 12428, 1462, 172, and 20, respectively. The limit of detection (LOD) was evaluated with a dilution series of 20, 10, 5, and 1 copies. A total of 10 independent real-time PCR reactions were performed for each of the G1 (2 runs) and G2 (1 run) petunias. The LOD was defined as the lowest copy number that was detected positively in all of the 10 reactions (ENGL, 2015).

## Results and Discussion

### PCR Analyses of Market Samples

After testing a large number of different petunia varieties sold on the market, the official control laboratories revealed a large set of PCR screening results^3^. Because of the regionally organized market surveillance in Germany, some varieties were tested by more than one official laboratory. However, identical PCR results were obtained which already indicated a similar genetic modification present in the different petunia varieties (**Table 1**). Based on these results, one group of varieties could be clearly linked to the GM petunia construct described by Meyer et al. (1987), similar to the Finnish finding of GM petunia on the market (Bashandy and Teeri, 2017). The plants predominantly show orange flowers but also other flower colors and are here assigned as G1 petunia (**Figures 1A, B**). Another small group of varieties with purple flowers contains several different GM elements and additional elements not characteristic for the G1 construct. These varieties are expressing a petunia flavonoid 3’5’ hydroxylase (F3’5’H) and are sorted in another group here assigned as G2 petunia (**Figures 1C, D**). In detail, G1 and G2 petunia varieties were positive in real-time PCR tests for P-35S and the P-nos/nptII construct (**Table 1**). The detected P-nos/nptII construct confirmed that P-nos is the promoter element of the nptII gene in both groups. In addition, G1 varieties were positive for T-35S and T-ocs while G2 petunias were negative for these elements (data not shown). Another obvious difference of G2 petunias was the presence of a T-nos element in contrast to the G1 varieties (**Table 1**). Interestingly, some petunia varieties with red or purple flowers (e.g. Classic Red, Johnny Flame and Pegasus Purple) were tested negative in the element- and construct-specific PCRs and therefore are not GM.

**Figure 2:**
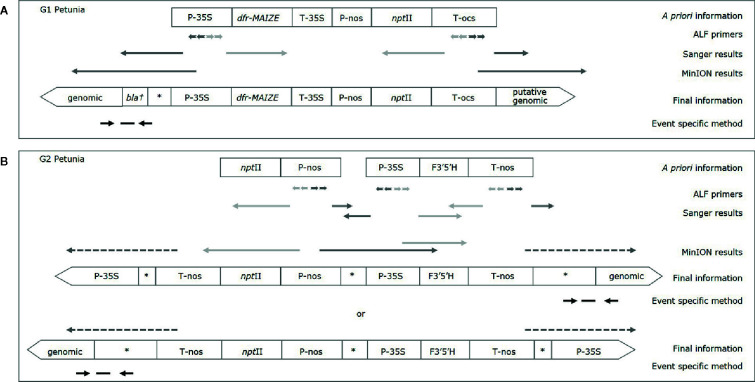
Characterization of G1 **(A)** and G2 **(B)** petunias. *A priori* information is based on (real-time) PCR results and available literature, final information is based on Sanger and MinION sequencing. Light grey arrows indicate enrichment into known sequence; dark grey arrows enrichment into unknown sequence, dashed arrows indicate a putative orientation. Event specific primers and probe are indicated by black arrows and a horizontal dash, respectively; an asterisk indicates a generic GM sequence (e.g. plasmid, vector); the dagger represents a putative partial sequence.

In order to connect more elements to each other, larger amplicons were generated for both groups by using the downstream primer of one element, and the upstream primer of another. The resulting amplicons were Sanger sequenced and analyzed by BLAST against GenBank. The element order and the presence of the insert containing the dfr-MAIZE-encoding gene in G1 and of the insert containing the F3’5’H-encoding gene in G2 petunia could be confirmed (**Figures 2A, B**). In addition, another combination of elements (P-nos/nptII) was identified in G2 by PCR tests, but it was not possible to link this combination to the F3’5’H-containing insert.

Sequences derived from G1 amplicons matched the sequence data available in GenBank for variety African Sunset (accession number KY964325.1) and the data determined recently for several petunia market samples (Fraiture et al., 2019). They also match the EUginius database entry for the MPI event RL01-17^8^ and event RL01-24 (sequence data provided by Meyer). For molecular characterization of these events, Meyer et al. had determined the sequence of plasmids pGS/MDF-17 (event RL01-17) and pGS/MDF-24 (event RL01-24). The sequence data encompass the complete sequence of the construct present in these events including the 5’- and 3’- regions of the petunia genome (**Supplementary Files 1** and **2**). The sequence of the element T-ocs is truncated in GM event RL01-17. Accordingly, PCR tests using DNA extracts from these samples would be negative when using a T-ocs element-specific method (Debode et al., 2013).

**Figure 3:**
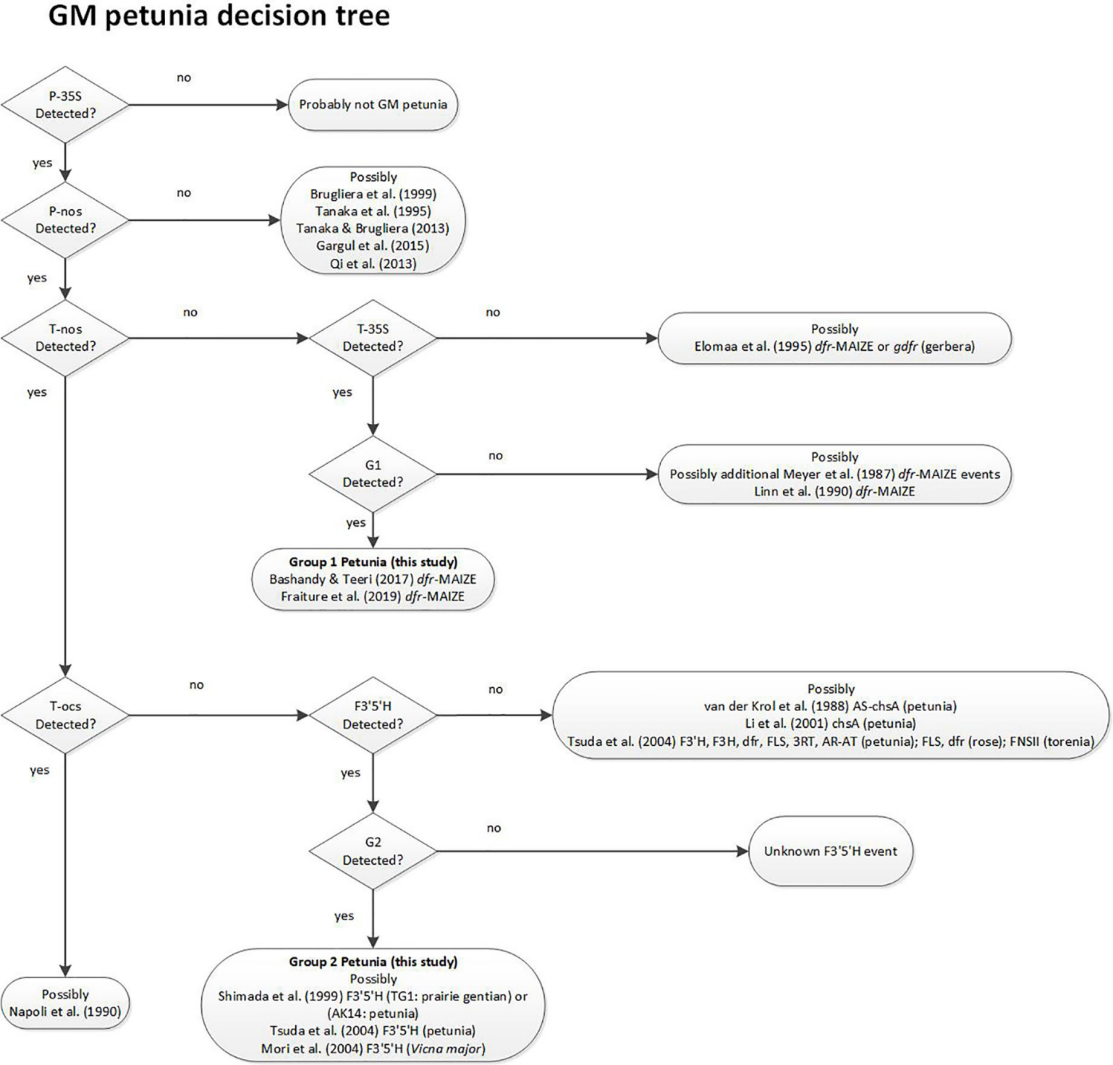
Decision Support System (DSS) for stepwise assignment of GM petunias to the potential origin of the construct. References describing the respective construct developments for GM petunias are indicated. Decision points are based on PCR test results.

Based on the results in the PCR tests for presence of the different elements and on the data available in the relevant literature, a decision support system (DSS) was developed to identify the most likely origin of an unassigned GM-positive petunia sample and provides additional relevant literature (**Figure 3**). The DSS helps to find literature references that potentially link to information regarding the event that was found. Decision criteria are based on some of the elements that were tested in this work. The suggested literature is only selective reading, and not a complete overview.

The DSS suggests that G1 varieties were developed by using a construct described by Meyer et al. (1987), as already substantiated earlier (Bashandy and Teeri, 2017). For G2 petunia the real-time PCR screening results and the Sanger sequence data obtained from amplicons suggested that between the P-35S and T-nos regulatory elements a DNA sequence identical to the petunia F3’5’H encoding gene (GenBank accession D14588.1) is present (**Figure 2B**). According to the DSS it is assumed that G2 petunias could originate from two different constructs (AK14 or pCGP1392) developed in Asia (Shimada et al., 1999; Tsuda et al., 2004).

### Determination of the 5’ and 3’ Flanking Regions in G1 and G2 Petunia

The gene walking-like ALF approach was performed to obtain construct-plant transition DNA sequence data for G1 and G2 petunia. Transition data were found for both groups, as well as further unexpected construct information in G2 (**Figures 2A, B**). To initiate enrichment in upstream and downstream directions several elements were selected. Initially, all ALF reaction mixtures were Sanger-sequenced with the element-specific ALF primers. The obtained sequences were analyzed by BLAST against GenBank and an in-house GM elements sequence database. Results largely confirmed the *a priori* known construct information. Additionally, an unexpected connection between P-nos and P-35S present in G2 in opposite orientations was found. The reads generated by Sanger sequencing were too short to reach clearly into the petunia genome, and therefore the ALF procedure was combined with the MinION sequencing approach to generate longer reads. Out of the initial 10 ALF reactions six were selected for MinION sequencing (**Table 3**).

**Figure 4:**
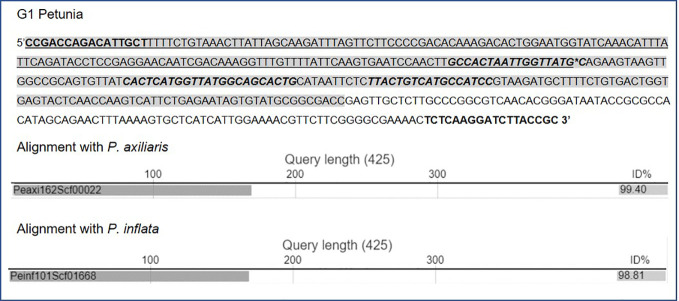
Sequence data of the junction region present in G1 petunia as determined by MinION sequencing and confirmed by Sanger sequencing (sequencing primers in bold letters). The junction site is indicated (*). The shaded sequence part in G1 shows 100% identity to the sequence recently reported by Fraiture et al. (2019) including their predicted junction site. Underlined are sequences with identity to *Petunia* ssp. sequences (Solgenomics database; alignments shown below sequence). Location of G1 primers and probe are bold italic.

For G1 petunia, the genome-oriented ALF reactions, upstream of P-35S and downstream of T-ocs, were sequenced using the MinION technology (**Figure 2A**). The sequences obtained by P-35S upstream enrichment matched for the first ~1,000 nt the putative bla/P-35S transition described by Bashandy and Teeri (2017; NCBI accession number KY964325). The remaining sequence did not show homology with any known GM element or event. BLAST analysis against petunia genomic sequences deposited in the Solgenomics database^9^ indicated the transition into the petunia genome as it showed high identity to *P. axillaris* and *P. inflata* sequences (**Figure 4**). The sequences downstream of T-ocs showed a larger variation and were therefore deemed less suitable for designing an event-specific method. For this reason, these sequences were not checked for homology with the petunia draft genome in the Solgenomics database, although the BLAST analysis did reveal homology with putative genomic DNA sequences (not shown).

The obtained sequence for the event-spanning region of G1 petunia was aligned to the recently published MinION derived raw sequence data (Fraiture et al., 2019). The alignment revealed 100% identity between the two sequences thereby confirming this region to represent the junction site between the petunia genome and the transgene construct present in G1 petunias (**Supplementary File 3**). The G1 event-spanning region was also aligned against the pGS/MDF-17 (line RL01-17) and pGS/MDF-24 (line RL-024) sequences, respectively. Results showed that G1 petunias contain a shorter bla (AmpR) sequence and a different transition sequence, thereby confirming that G1 petunias are not derived from MPI lines RL01-17 or RL01-24.

**Figure 5:**
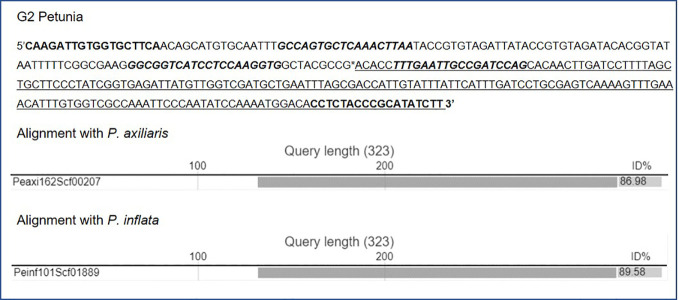
Sequence data of the junction region present in G2 petunia as determined by MinION sequencing and confirmed by Sanger sequencing (sequencing primers in bold letters). Underlined are sequences with identity to *Petunia* ssp. sequences (Solgenomics database; alignments shown below sequence). Location of G2 primers and probe are bold italic.

For G2, four ALF reactions were selected for MinION sequencing to gain information on the composition of the insert as well as at least one event-spanning region (**Table 3**). As shown in **Figure 2B**, a genomic transition was found downstream of T-nos, the P-nos/vector/P-35S connection was confirmed, and an additional T-nos element was found. This additional T-nos was found directly downstream of the P-nos/nptII part and is most likely the functional terminator of the nptII expression cassette. From this, the presence of a construct was concluded with two coding sequences in opposite directions, one for nptII and one for F3’5’H, with the promotors in the middle and a T-nos terminator at either end. This organization of elements also explained why two different sets of sequences were found downstream of T-nos. Interestingly, our present data could not indicate on which side of the construct the two different parts are located, as our sequence information did not traverse the T-nos sequence but the obtained results indicated two potential organizations of elements. One set of T-nos downstream sequences revealed an unidentified sequence, a generic plasmid sequence and a partial P-35S element identifying a so far unknown part of the G2 genetic modification. However, this sequence could not be linked to petunia, i.e. no sequence similarity was observed with the petunia genomes in the Solgenomics database and was therefore not investigated further. The other T-nos downstream sequence showed that the first ~80 nt constitute T-nos, followed by a stretch of ~150 nt generic plasmid sequence and ~1,300 nt of unidentified sequence. Megablast analysis (NCBI) of this unidentified region showed that the first ~600 nt had approximately 98% identity with multiple vector sequences. The remaining 700 nt of the sequence did not show homology with any known GM element. This part was therefore examined with BLAST against the petunia draft genomes *P. axillaris* and *P. inflata* in the Solgenomics database, which revealed over 87-93% sequence identity with petunia genomic sequences. The in-depth search for homologies to known sequences deposited in NCBI GenBank and the Solgenomics database showed that the sequence between T-nos and the identified petunia sequence is only partly homologous to petunia and other higher plants. This suggests that this region consists of unknown rearrangements, possibly caused by the insertion of the GM cassette. This region was identified as representing the junction region in G2 petunia and was confirmed using Sanger sequencing prior to the design of the event-specific real-time PCR method (**Figure 5**; **Supplementary File 3**).

### Event-Specific Real-Time PCR Detection Methods

To confirm the correctness of the sequences of the putative junction sites in G1 (P-35S upstream sequence, **Figure 2A**) and G2 (T-nos downstream sequence, **Figure 2B**) primers were designed approximately 250 nt up- and downstream. The resulting amplicons were Sanger sequenced, and the data were BLAST analyzed and used for designing primers and a probe for the event-specific real-time PCR methods.

The designed event-specific detection methods (**Figures 2A, B**) were first tested with DNA extracted from different G1 and G2 representatives and from non-GM petunias (see Chapter 2). DNA extracted from Viva Orange Vein (G1) and Raspberry Blast (G2) were positive in the respective G1 and G2 event-specific real-time PCR tests. To validate the method performance characteristics, the PCR efficiency, the correlation coefficient (*R^2^*) and the LOD were determined (**Table 4**). The data obtained were in compliance with the requirements described by ENGL (2015), although PCR efficiency and the correlation coefficient are not necessarily required for qualitative methods.

**Table 4:** Efficiency, *R^2^* and LOD for G1 and G2 petunia event-specific real-time PCR methods.

Group	Sample	Efficiency (%)	*R^2^*	LOD
G1 petunia	African Sunset	103.3	0.998	10 cp (19/19)*
	Go!Tunia Orange	96.9	0.994	10 cp (20/20)*
G2 petunia	Raspberry Blast	93.5	0.995	10 cp (10/10)
	Mini Blast Rose	90.1	0.998	5 cp (10/10)

*For G1 DNAs, two dilution series were tested (and one failure test for variety African Sunset).

As a next step, the partially validated event-specific real-time PCR methods were tested for specificity using genomic DNAs extracted from different petunia varieties. In an interlaboratory comparison study with four participating laboratories, DNAs extracted from samples taken from different market places during the inspections in 2017 were tested (**Table 1**). All tested petunia varieties with orange flower colors were negative for the element T-nos, but clearly positive (average Cq of 25.9 ± 1.7) if tested with the G1 event-specific real-time PCR system. Likewise, all pink flowering petunia varieties were positive for T-nos and were clearly detected by the G2 event-specific real-time PCR (average Cq of 26.6 ± 1.2). Tests using the G1 and G2 event-specific real-time PCR methods were negative when DNA extracted from the materials of events RL01-17 and RL01-24 was examined (**Table 1**), indicating that events MPI-17 (235/1-17 or RL01-17) and RL01-24 (or 235/1-24) can be excluded as progenitors of the G1-derived cultivars possibly deregulated (USDA-APHIS, 2019).

## Conclusions

Putative GM petunias were screened with a set of element- and construct-specific real-time PCR methods. In all cases, one or more of these tests were positive, thereby confirming these petunias to be GM. Furthermore, the screening revealed that two groups of GM petunias were sold on the market. G1 petunias correspond to the dfr-MAIZE construct described by Meyer et al. (1987). G2 petunias containing the F3’5’H construct could yet not be linked to a research publication or a sequence database. The developed DSS provides guidance as to how to stepwise assign GM petunias to the potential origin of the construct based on available literature.

The event-spanning genomic junction regions could be amplified for the orange-flowering G1 variety African Sunset and the purple-flowering G2 variety Raspberry Blast. Using MinION and Sanger sequencing these junction regions were determined. The sequence comparison of the event-spanning regions confirmed, that G1 petunias are not derived from MPI lines RL01-17 or RL01-24.

Based on the sequence data of the G1 and G2 varieties, event-specific real-time PCR methods were developed and in-house validated. They both fulfill the established criteria regarding specificity, efficiency, linearity, and LOD (ENGL, 2015).

After validation, these methods could be used to unambiguously assign diverse varieties to be G1 or G2 petunias. These tests again confirm, that the MPI event RL01-17 used in the German field trial in 1991 is not the origin of the orange flowering petunia varieties sold on the market.

The event-specific real-time PCR methods will allow rapid detection and identification of GM petunias by competent authorities and breeders who want to test breeding lines either for absence of GM material or presence of the GM events possibly deregulated in the US.

## Data Availability Statement

The datasets presented in this study can be found in online repositories. The names of the repository/repositories and accession number(s) can be found in the article/**Supplementary Material**.

## Author Contributions

MV, TP, JD, EK, and IS contributed to the design and implementation of the research. MV, TP, AB, CB-N, OG, and BP designed and performed the experiments. MV, TP, AB, CB-N, JD, OG, BP, IS, and LG analyzed the data and interpreted the results. MV, TP, JB, JD, EK, IS, LG, and AS contributed to writing the manuscript. All authors contributed to the article and approved the submitted version.

## Funding

WFSR received funding from the Human Environment and Transport Inspectorate (Inspectie Leefomgeving en Transport, ILT) for screening of GM petunias, and from ILT and BVL for the development of the here described methods.

## Disclaimer

The views or positions expressed in this publication do not necessarily represent in legal terms the official position of the institutions or organization the authors work for.

## Conflict of Interest

The authors declare that the research was conducted in the absence of any commercial or financial relationships that could be construed as a potential conflict of interest.

## Footnotes

1
https://geenitekniikanlautakunta.fi/en/article/-/asset_publisher/luvattomia-gm-petunioita-suomen-markkinoilla

2
https://zag.bvl.bund.de/freisetzungen/detail.jsf?dswid=5418&dsrid=613&azLink=6786-01-0001

3
https://securite-alimentaire.public.lu/dam-assets/fr/professionnel/OGM/Organismes-genetiquement-modifies-ornementaux-_non-alimentaire_/Petunias/Trade-names-confirmed-as-GM-2019-03-11.pdf

4
https://www.aphis.usda.gov/aphis/ourfocus/biotechnology/brs-news-and-information/2017_brs_news/petunia_overview

5
https://cogem.net/en/publication/unauthorised-gm-garden-petunia-varieties-with-orange-flowers/

6
http://www.zkbs-online.de/ZKBS/SharedDocs/Downloads/01_Allgemeine%20Stellungnahmen/04%20Pflanzen/Petunien_gentechnisch_ver%C3%A4nderte_2017.pdf;jsessionid=B5E31096316F53E2AD69FC398ABA271E.1_cid350?:blob=publicationFile&v=9

7
https://cvalues.science.kew.org

8
http://www.euginius.eu/euginius/pages/gmo_detail.jsf?gmoname=RL01-17

9
https://solgenomics.net/
