# Supplementary material for: Molecular Characterization and Event-Specific Real-Time PCR Detection of Two Dissimilar Groups of Genetically Modified Petunia (Petunia x hybrida) Sold on the Market
Source: Front Plant Sci. 2020 Jul 14;11:1047. doi: 10.3389/fpls.2020.01047 (PMC7372090; doi:10.3389/fpls.2020.01047)
Supplement: Supplementary file 1 [file DataSheet_1.pdf]

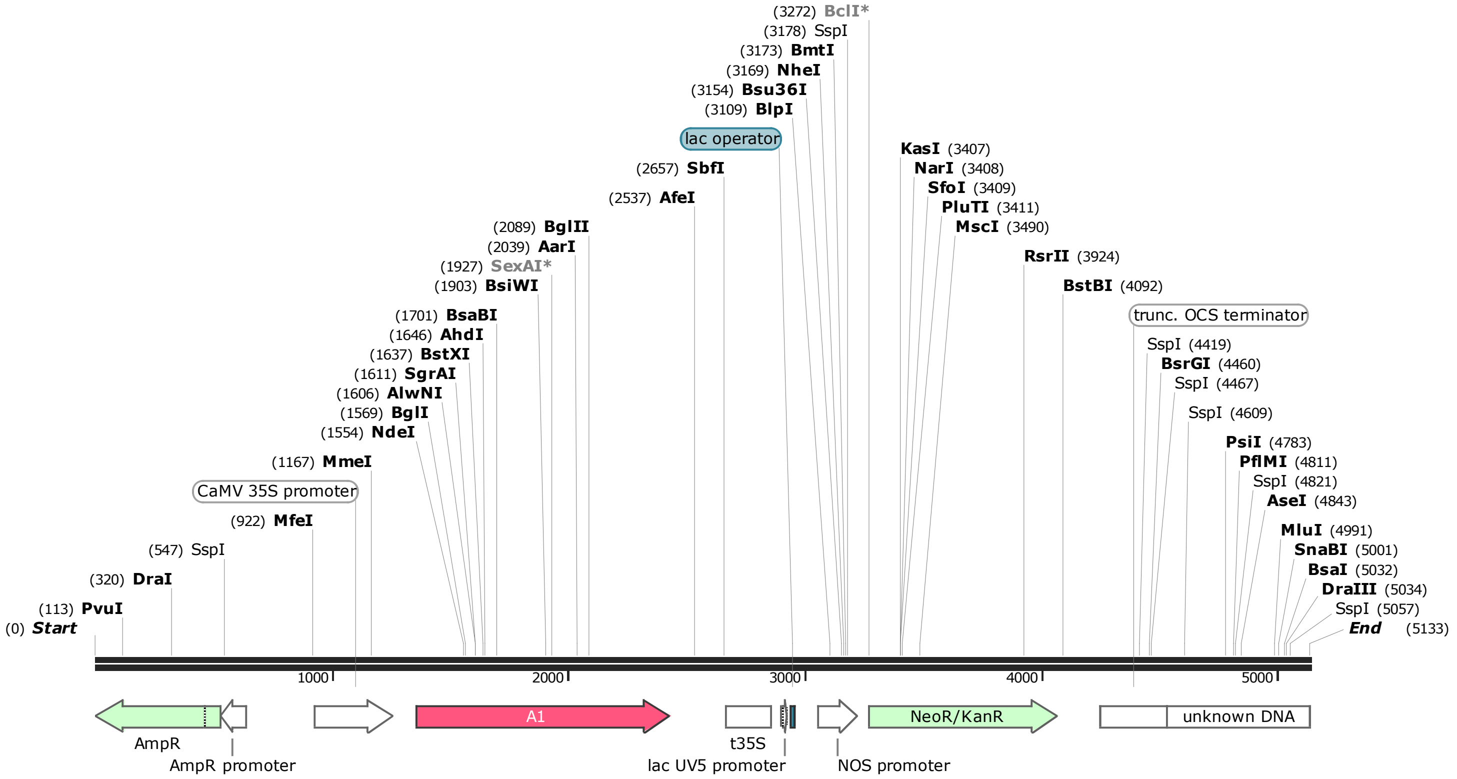

LGL_Contig_pGSMDF-17
5133 bp

Start (0)

5′ CGTTCGTCGTTTGGTAGGGTTTCATTCAGTTCCGGTTTCCCAAGGATCAAGGCGGAGTTACATGATCCCCCATGTTGGGCAAAAAAGCG 90
3′ GCAAGCAGCAAACCATCCCAAAGTAAGTCAAGGCCAAAGGGTTCCTAGTTCCGCCTCAATGTACTAGGGGGTACAACCCGTTTTTTCGC

175 170 165 160 155 150
E D N P L T E N L E P K G L I L A S N C S G G M N P L F A
AmpR

PvuI

GTTAGCTCCTTCGGTCCTCCGATCGTTGTCAGAAGTAAGTTGGCCGCAGTGTTATCACTCATGGTTATGGCAGCACTGCATAATTCTCTT 180
CAATCGAGGAAGCCAGGAGGCTAGCAACAGTCTTCATTCAACCGGCGTCACAATAGTGAGTACCAATACCGTCGTGACGTATTAAGAGAA

145 140 135 130 125 120
T L E K P G G I T T L L L N A A T N D S M T I A A S C L E R
AmpR

ACTGTCATGCCATCCGTAAGATGCTTTTCTGTGACTGGTGAGTACTCAACCAAGTCATTCTGAGAATAGTGTATGCGGCGACCGAGTTGC 270
TGACAGTACGGTAGGCATTCTACGAAAAGACACTGACCACTCATGAGTTGGTTCAGTAAGACTCTTATCACATACGCCGCTGGCTCAACG

115 110 105 100 95 90
V T M G D T L H K E T V P S Y E V L D N Q S Y H I R R G L Q
AmpR

DraI

TCTTGCCCGGCGTCAACACGGGATAATACCGCGCCACATAGCAGAACTTTAAAAGTGCTCATCATTGGAAAACGTTCTTCGGGGCGAAAA 360
AGAACGGGCCGCAGTTGTGCCCTATTATGGCGCGGTGTATCGTCTTGAAATTTTCACGAGTAGTAACCTTTTGCAAGAAGCCCCGCTTTT

85 80 75 70 65 60
E Q G A D V R S L V A G C L L V K F T S M M P F R E E P R F
AmpR

CTCTCAAGGATCTTACCGCTGTTGAGATCCAGTTCGATGTAACCCACTCGTGCACCCAACTGATCTTCAGCATCTTTTACTTTCACCAGC 450
GAGAGTTCCTAGAATGGCGACAACTCTAGGTCAAGCTACATTGGGTGAGCACGTGGGTTGACTAGAAGTCGTAGAAAATGAAAGTGGTCG

55 50 45 40 35 30
S E L I K G S N L D L E I Y G V R A G L Q D E A D K V K V L
AmpR

GTTTCTGGGTGAGCAAAAACAGGAAGGCAAAATGCCGCAAAAAAGGGAATAAGGGCGACACGGAAATGTTGAATACTCATACTCTTCCTT 540
CAAAGACCCACTCGTTTTTGTCCTTCCGTTTTACGGCGTTTTTTCCCTTATTCCCGCTGTGCCTTTACAACTTATGAGTATGAGAAGGAA

25 20 15 10 5 1
T E P H A F V P L C F A A F F P I L A V R F H Q I S M
signal sequence
AmpR
AmpR promoter

SspI

TTTCAATATTATTGAAGCATTTATCAGGGTTATTGTCTCATGAGCGGATACATATTTGAATGTATTTAGAAAAATAAACAAATAGGGGTT 630
AAAGTTATAATAACTTCGTAAATAGTCCCAATAACAGAGTACTCGCCTATGTATAAACTTACATAAATCTTTTTATTTGTTTATCCCCAA

AmpR promoter

CCGCGCACATTTCCCCGAAAAGTGCCACCTGACGTCTAAGAAACCATTATTATCATGACATTAACCTATAAAAATAGGCGTATCACGAGG 720
GGCGCGTGTAAAGGGGCTTTTCACGGTGGACTGCAGATTCTTTGGTAATAATAGTACTGTAATTGGATATTTTTATCCGCATAGTGCTCC

AmpR promoter

CCCTTTCGTCTTCAAGAATTCCCATGGAGTCAAAGATTCAAATAGAGGACCTAACAGAACTCGCCGTAAAGACTGGCGAACAGTTCATAC 810
GGGAAAGCAGAAGTTCTTAAGGGTACCTCAGTTTCTAAGTTTATCTCCTGGATTGTCTTGAGCGGCATTTCTGACCGCTTGTCAAGTATG

AGAGTCTCTTACGACTCAATGACAAGAAGAAAATCTTCGTCAACATGGTGGAGCACGACACGCTTGTCTACTCCAAAAATATCAAAGATA 900
TCTCAGAGAATGCTGAGTTACTGTTCTTCTTTTAGAAGCAGTTGTACCACCTCGTGCTGTGCGAACAGATGAGGTTTTTATAGTTTCTAT

MfeI

CAGTCTCAGAAGACCAAAGGGCAATTGAGACTTTTCAACAAAGGGTAATATCCGGAAACCTCCTCGGATTCCATTGCCCAGCTATCTGTC 990

GTCAGAGTCTTCTGGTTTCCCGTTAACTCTGAAAAGTTGTTTCCCATTATAGGCCTTTGGAGGAGCCTAAGGTAACGGGTCGATAGACAG

CaMV 35S promoter

ACTTTATTGTGAAGATAGTGGAAAAGGAAGGTGGCTCCTACAAATGCCATCATTGCGATAAAGGAAAGGCCATCGTTGAAGATGCCTCTG 1080

TGAAATAACACTTCTATCACCTTTTCCTTCCACCGAGGATGTTTACGGTAGTAACGCTATTTCCTTTCCGGTAGCAACTTCTACGGAGAC

CaMV 35S promoter

MmeI

CCGACAGTGGTCCCAAAGATGGACCCCCACCCACGAGGAGCATCGTGGAAAAAGAAGACGTTCCAACCACGTCTTCAAAGCAAGTGGATT 1170

GGCTGTCACCAGGGTTTCTACCTGGGGGTGGGTGCTCCTCGTAGCACCTTTTTCTTCTGCAAGGTTGGTGCAGAAGTTTCGTTCACCTAA

CaMV 35S promoter

GATGTGATATCTCCACTGACGTAAGGGATGACGCACAATCCCACTATCCTTCGCAAGACCCTTCCTCTATATAAGGAAGTTCATTTCATT 1260

CTACACTATAGAGGTGACTGCATTCCCTACTGCGTGTTAGGGTGATAGGAAGCGTTCTGGGAAGGAGATATATTCCTTCAAGTAAAGTAA

CaMV 35S promoter

TGGAGAGGACAGGGTACCCGGGGATCCTCTAGAGAATTCCAGCTGCTCACTCAGTCCTGCGCAAGAGCTCGCTCTCGGAGAAAAAAACGC 1350

ACCTCTCCTGTCCCATGGGCCCCTAGGAGATCTCTTAAGGTCGACGAGTGAGTCAGGACGCGTTCTCGAGCGAGAGCCTCTTTTTTTGCG

CaMV 35S promoter

GGGAGGCGATAATGGAGGGAGGTGCCGGTGCGAGCGAGAAAGGGACGGTGCTGGTCACGGGGGCGTCGGGCTTCGCCGGCTCCTGGCTCG 1440

CCCTCCGCTATTACCTCCCTCCACGGCCACGCTCGCTCTTTCCCTGCCACGACCAGTGCCCCCGCAGCCCGAAGCGGCCGAGGACCGAGC

1 5 10 15 20 25

M E G G A G A S E K G T V L V T G A S G F A G S W L

A1

TCATGAAGCTCCTCCAGGCCGGCTACACCGTCCGGGCGACCGTGCGCGATCCCGCGAACGTTGGGAAGACGAAGCCATTGATGGACCTTC 1530

AGTACTTCGAGGAGGTCCGGCCGATGTGGCAGGCCCGCTGGCACGCGCTAGGGCGCTTGCAACCCTTCTGCTTCGGTAACTACCTGGAAG

30 35 40 45 50 55

V M K L L Q A G Y T V R A T V R D P A N V G K T K P L M D L

A1

NdeI BglI AlwNI SgrAI

CCGGAGCAACGGAGCGCCTGTCCATATGGAAAGCCGACCTGGCGGAGGAAGGCAGCTTCCACGACGCCATCAGGGGCTGCACCGGCGTCT 1620

GGCCTCGTTGCCTCGCGGACAGGTATACCTTTCGGCTGGACCGCCTCCTTCCGTCGAAGGTGCTGCGGTAGTCCCCGACGTGGCCGCAGA

60 65 70 75 80 85

P G A T E R L S I W K A D L A E E G S F H D A I R G C T G V

A1

BstXI AhdI BsaBI

TCCACGTCGCCACGCCCATGGACTTCCTGTCCAAAGACCCTGAGAATGAGGTAATCAAGCCGACGGTGGAAGGGATGATAAGCATCATGC 1710

AGGTGCAGCGGTGCGGGTACCTGAAGGACAGGTTTCTGGGACTCTTACTCCATTAGTTCGGCTGCCACCTTCCCTACTATTCGTAGTACG

90 95 100 105 110 115

F H V A T P M D F L S K D P E N E V I K P T V E G M I S I M

A1

GGGCATGCAAGGAGGCCGGCACCGTGCGGCGCATCGTCTTCACTTCCTCCGCCGGGACGGTCAACCTGGAGGAGCGGCAGAGGCCCGTCT 1800

CCCGTACGTTCCTCCGGCCGTGGCACGCCGCGTAGCAGAAGTGAAGGAGGCGGCCCTGCCAGTTGGACCTCCTCGCCGTCTCCGGGCAGA

120 125 130 135 140 145

R A C K E A G T V R R I V F T S S A G T V N L E E R Q R P V

A1

ACGACGAGGAAAGCTGGACCGACGTCGACTTCTGCCGTCGCGTCAAGATGACAGGATGGATGTACTTCGTGTCTAAAACCCTGGCGGAGA 1890

TGCTGCTCCTTTCGACCTGGCTGCAGCTGAAGACGGCAGCGCAGTTCTACTGTCCTACCTACATGAAGCACAGATTTTGGGACCGCCTCT

150 155 160 165 170 175

Y D E E S W T D V D F C R R V K M T G W M Y F V S K T L A E

A1

BsiWI SexAI*

AGGCGGCCCTGGCGTACGCGGCGGAGCACGGCCTGGACCTGGTCACCATCATCCCGACGCTCGTGGTCGGCCCGTTCATCAGCGCGTCCA 1980

TCCGCCGGGACCGCATGCGCCGCCTCGTGCCGGACCTGGACCAGTGGTAGTAGGGCTGCGAGCACCAGCCGGGCAAGTAGTCGCGCAGGT

180 185 190 195 200 205

K A A L A Y A A E H G L D L V T I I P T L V V G P F I S A S

A1

AarI

TGCCGCCCAGCCTCATCACCGCGCTGGCGCTCATCACGGGGAACGCGCCGCACTACTCGATCCTCAAGCAGGTGCAGCTCATCCACCTCG 2070

ACGGCGGGTCGGAGTAGTGGCGCGACCGCGAGTAGTGCCCCTTGCGCGGCGTGATGAGCTAGGAGTTCGTCCACGTCGAGTAGGTGGAGC

210 215 220 225 230 235

M P P S L I T A L A L I T G N A P H Y S I L K Q V Q L I H L

A1

BglII

ACGACCTCTGCGACGCCGAGATCTTCCTCTTCGAGAACCCGGCCGCGGCCGGGCGCTACGTTTGCTCCTCGCACGACGTCACCATCCACG 2160

TGCTGGAGACGCTGCGGCTCTAGAAGGAGAAGCTCTTGGGCCGGCGCCGGCCCGCGATGCAAACGAGGAGCGTGCTGCAGTGGTAGGTGC

240 245 250 255 260 265

D D L C D A E I F L F E N P A A A G R Y V C S S H D V T I H

A1

GCCTCGCCGCCATGCTCAGGGATAGGTACCCCGAGTACGACGTCCCGCAGAGGTTCCCCGGGATCCAGGACGACCTCCAGCCGGTGCGCT 2250

CGGAGCGGCGGTACGAGTCCCTATCCATGGGGCTCATGCTGCAGGGCGTCTCCAAGGGGCCCTAGGTCCTGCTGGAGGTCGGCCACGCGA

270 275 280 285 290 295

G L A A M L R D R Y P E Y D V P Q R F P G I Q D D L Q P V R

A1

TCTCGTCCAAGAAGCTCCAGGACCTCGGGTTCACCTTCAGGTACAAGACGCTGGAGGACATGTTCGACGCCGCCATCCGGACTTGCCAGG 2340

AGAGCAGGTTCTTCGAGGTCCTGGAGCCCAAGTGGAAGTCCATGTTCTGCGACCTCCTGTACAAGCTGCGGCGGTAGGCCTGAACGGTCC

300 305 310 315 320 325

F S S K K L Q D L G F T F R Y K T L E D M F D A A I R T C Q

A1

AGAAGGGCCTCATCCCCCTCGCCACTGCCGCCGGAGGGGACGGCTTTGCCTCGGTGCGCGCACCCGGCGAGACGGAGGCGACGATTGGCG 2430

TCTTCCCGGAGTAGGGGGAGCGGTGACGGCGGCCTCCCCTGCCGAAACGGAGCCACGCGCGTGGGCCGCTCTGCCTCCGCTGCTAACCGC

330 335 340 345 350 355

E K G L I P L A T A A G G D G F A S V R A P G E T E A T I G

A1

CTTAGGCAACGATCCCCCGGCTCTCCCCGTCGATATGATGCAATCAGCTATCTATCTCTTGTTTGCCAAAAAAAATAAGGGAGGTCTTGG 2520

GAATCCGTTGCTAGGGGGCCGAGAGGGGCAGCTATACTACGTTAGTCGATAGATAGAGAACAAACGGTTTTTTTTATTCCCTCCAGAACC

A *

A1

AfeI

CATACTCGATYTAGAGCGCTTTGCAAGAGCGTTAAGGCTTAGATGACTATGGTTACGATGGACGAATAGAGACAAAGCATGGACTGGGTT 2610

GTATGAGCTARATCTCGCGAAACGTTCTCGCAATTCCGAATCTACTGATACCAATGCTACCTGCTTATCTCTGTTTCGTACCTGACCCAA

SbfI

GCAATTAAAAAAAAAAAAAAAAAGGAATTCTCTAGAGTCGACCTGCAGGCATGCCCGCTGAAATCACCAGTCTCTCTCTACAAATCTATC 2700

CGTTAATTTTTTTTTTTTTTTTTCCTTAAGAGATCTCAGCTGGACGTCCGTACGGGCGACTTTAGTGGTCAGAGAGAGATGTTTAGATAG

t35S

TCTCTCTATAATAATGTGTGAGTAGTTCCCAGATAAGGGAATTAGGGTTCTTATAGGGTTTCGCTCATGTGTTGAGCATATAAGAAACCC 2790

AGAGAGATATTATTACACACTCATCAAGGGTCTATTCCCTTAATCCCAAGAATATCCCAAAGCGAGTACACAACTCGTATATTCTTTGGG

t35S

TTAGTATGTATTTGTATTTGTAAAATACTTCTATCAATAAAATTTCTAATTCCTAAAACCAAAATCCAGGGGTACCGAGCTCGAATTCTC 2880

AATCATACATAAACATAAACATTTTATGAAGATAGTTATTTTAAAGATTAAGGATTTTGGTTTTAGGTCCCCATGGCTCGAGCTTAAGAG

t35S

ACTCATTAGGCACCCCAGGCTTTACACTTTATGCTTCCGGCTCGTATAATGTGTGGAATTGTGAGCGGATAACAATTTCACACAGGAAAC 2970
TGAGTAATCCGTGGGGTCCGAAATGTGAAATACGAAGGCCGAGCATATTACACACCTTAACACTCGCCTATTGTTAAAGTGTGTCCTTTG
-35 -10 lac operator
lac UV5 promoter

AGGATCATGAGCGGAGAATTAAGGGAGTCACGTTATGACCCCCGCCGATGACGCGGGACAAGCCGTTTTACGTTTGGAACTGACAGAACC 3060
TCCTAGTACTCGCCTCTTAATTCCCTCAGTGCAATACTGGGGGCGGCTACTGCGCCCTGTTCGGCAAAATGCAAACCTTGACTGTCTTGG
NOS promoter

BlpI
GCAACGTTGAAGGAGCCACTCAGCCGCGGGTTTCTGGAGTTTAATGAGCTAAGCACATACGTCAGAAACCATTATTGCGCGTTCAAAAGT 3150
CGTTGCAACTTCCTCGGTGAGTCGGCGCCCAAAGACCTCAAATTACTCGATTCGTGTATGCAGTCTTTGGTAATAACGCGCAAGTTTTCA
NOS promoter

Bsu36I **NheI** **BmtI** SspI
CGCCTAAGGTCACTATCAGCTAGCAAATATTTCTTGTCAAAAATGCTCCACTGACGTTCCATAAATTCCCCTCGGTATCCAATTAGAGTC 3240
GCGGATTCCAGTGATAGTCGATCGTTTATAAAGAACAGTTTTTACGAGGTGACTGCAAGGTATTTAAGGGGAGCCATAGGTTAATCTCAG
NOS promoter

BclI*
TCATATTCACTCTCAATCCAGATCCGGCCCATGATCATGTGGATTGAACAAGATGGATTGCACGCAGGTTCTCCGGCCGCTTGGGTGGAG 3330
AGTATAAGTGAGAGTTAGGTCTAGGCCGGGTACTAGTACACCTAACTTGTTCTACCTAACGTGCGTCCAAGAGGCCGGCGAACCCACCTC
1 5 10 15
M W I E Q D G L H A G S P A A W V E
NeoR/KanR

KasI **NarI** **SfoI** **PluTI**
AGGCTATTCGGCTATGACTGGGCACAACAGACAATCGGCTGCTCTGATGCCGCCGTGTTCCGGCTGTCAGCGCAGGGGCGCCCGGTTCTT 3420
TCCGATAAGCCGATACTGACCCGTGTTGTCTGTTAGCCGACGAGACTACGGCGGCACAAGGCCGACAGTCGCGTCCCCGCGGGCCAAGAA
20 25 30 35 40 45
R L F G Y D W A Q Q T I G C S D A A V F R L S A Q G R P V L
NeoR/KanR

MscI
TTTGTCAAGACCGACCTGTCCGGTGCCCTGAATGAACTGCAGGACGAGGCAGCGCGGCTATCGTGGCTGGCCACGACGGGCGTTCCTTGC 3510
AAACAGTTCTGGCTGGACAGGCCACGGGACTTACTTGACGTCCTGCTCCGTCGCGCCGATAGCACCGACCGGTGCTGCCCGCAAGGAACG
50 55 60 65 70 75
F V K T D L S G A L N E L Q D E A A R L S W L A T T G V P C
NeoR/KanR

GCAGCTGTGCTCGACGTTGTCACTGAAGCGGGAAGGGACTGGCTGCTATTGGGCGAAGTGCCGGGGCAGGATCTCCTGTCATCTCACCTT 3600
CGTCGACACGAGCTGCAACAGTGACTTCGCCCTTCCCTGACCGACGATAACCCGCTTCACGGCCCCGTCCTAGAGGACAGTAGAGTGGAA
80 85 90 95 100 105
A A V L D V V T E A G R D W L L L G E V P G Q D L L S S H L
NeoR/KanR

GCTCCTGCCGAGAAAGTATCCATCATGGCTGATGCAATGCGGCGGCTGCATACGCTTGATCCGGCTACCTGCCCATTCGACCACCAAGCG 3690
CGAGGACGGCTCTTTCATAGGTAGTACCGACTACGTTACGCCGCCGACGTATGCGAACTAGGCCGATGGACGGGTAAGCTGGTGGTTCGC
110 115 120 125 130 135
A P A E K V S I M A D A M R R L H T L D P A T C P F D H Q A
NeoR/KanR

AAACATCGCATCGAGCGAGCACGTACTCGGATGGAAGCCGGTCTTGTCGATCAGGATGATCTGGACGAAGAGCATCAGGGGCTCGCGCCA 3780
TTTGTAGCGTAGCTCGCTCGTGCATGAGCCTACCTTCGGCCAGAACAGCTAGTCCTACTAGACCTGCTTCTCGTAGTCCCCGAGCGCGGT
140 145 150 155 160 165
K H R I E R A R T R M E A G L V D Q D D L D E E H Q G L A P
NeoR/KanR

```
GCCGAACTGTTCGCCAGGCTCAAGGCGCGCATGCCCGACGGCGAGGATCTCGTCGTGACCCATGGCGATGCCTGCTTGCCGAATATCATG   3870
CGGCTTGACAAGCGGTCCGAGTTCCGCGCGTACGGGCTGCCGCTCCTAGAGCAGCACTGGGTACCGCTACGGACGAACGGCTTATAGTAC
 170        175        180        185        190        195
A  E  L  F  A  R  L  K  A  R  M  P  D  G  E  D  L  V  V  T  H  G  D  A  C  L  P  N  I  M
NeoR/KanR

RsrII
GTGGAAAATGGCCGCTTTTCTGGATTCATCGACTGTGGCCGGCTGGGTGTGGCGGACCGCTATCAGGACATAGCGTTGGCTACCCGTGAT   3960
CACCTTTTACCGGCGAAAAGACCTAAGTAGCTGACACCGGCCGACCCACACCGCCTGGCGATAGTCCTGTATCGCAACCGATGGGCACTA
 200        205        210        215        220        225
V  E  N  G  R  F  S  G  F  I  D  C  G  R  L  G  V  A  D  R  Y  Q  D  I  A  L  A  T  R  D
NeoR/KanR

ATTGCTGAAGAGCTTGGCGGCGAATGGGCTGACCGCTTCCTCGTGCTTTACGGTATCGCCGCTCCCGATTCGCAGCGCATCGCCTTCTAT   4050
TAACGACTTCTCGAACCGCCGCTTACCCGACTGGCGAAGGAGCACGAAATGCCATAGCGGCGAGGGCTAAGCGTCGCGTAGCGGAAGATA
 230        235        240        245        250        255
I  A  E  E  L  G  G  E  W  A  D  R  F  L  V  L  Y  G  I  A  A  P  D  S  Q  R  I  A  F  Y
NeoR/KanR

BstBI
CGCCTTCTTGACGAGTTCTTCTGARCGGGAaCTCTgGGGGTTCGAAATGACCGACCAAGCGACGCCCAAaCCTGCCATCACGAGrATTTC   4140
GCGGAAGAACTGCTCAAGAAGACTYGCCCTtGAGAcCCCCAAGCTTTACTGGCTGGTTCGCTGCGGGTTtGGACGGTAGTGCTCyTAAAG
 260        265
R  L  L  D  E  F  F  *
NeoR/KanR

GATTCCACCGCCGCCTTCTATGAAAaGGTTGGGCTTCGGAATCGTTTTCCGGGACGCCGGCTGGATGATCCTCCAGCGCGGGGATCTCAT   4230
CTAAGGTGGCGGCGGAAGATACTTTtCCAACCCGAAGCCTTAGCAAAAGGCCCTGCGGCCGACCTACTAGGAGGTCGCGCCCCTAGAGTA

GCTGGAGTTCTTCGCCCACCCCCTGCTTTAATGAGATATGCGAGACGCCTATGATCGCATGATATTTGCTTTCAATTCTGTTGTGCACGT   4320
CGACCTCAAGAAGCGGGTGGGGGACGAAATTACTCTATACGCTCTGCGGATACTAGCGTACTATAAACGAAAGTTAAGACAACACGTGCA
trunc. OCS terminator

TGTAAAAAACCTGAGCATGTGTAGCTCAGATCCTTACCGCCGGTTTCGGTTCATTCTAATGAATATATCACCCGTTACTATCGTATTTTT   4410
ACATTTTTTGGACTCGTACACATCGAGTCTAGGAATGGCGGCCAAAGCCAAGTAAGATTACTTATATAGTGGGCAATGATAGCATAAAAA
trunc. OCS terminator

SspI  BsrGI  SspI
ATGAATAATATTCTCCGTTCAATTTACTGATTGTACCCTACTACTTATATGTACAATATTAAAATGAAAACAATATATTGTGCTGAATAG   4500
TACTTATTATAAGAGGCAAGTTAAATGACTAACATGGGATGATGAATATACATGTTATAATTTTACTTTTGTTATATAACACGACTTATC
trunc. OCS terminator

GTTTATAGCGACATCTATGATAGAGCGCCACATGTTCATTGATTTTTGTTGCCCTCCATAAATGTATTTCATATATTGTATAAATTTTCA   4590
CAAATATCGCTGTAGATACTATCTCGCGGTGTACAAGTAACTAAAAACAACGGGAGGTATTTACATAAAGTATATAACATATTTAAAAGT
trunc. OCS terminator   unknown DNA

SspI
TATAATTATGTTTTTCAATATTTAAGACTTTAACGTCAACTGATATCTTAAACCTGTATAACTTTTAAGTCAACAAATATATTCTTTATA   4680
ATATTAATACAAAAAGTTATAAATTCTGAAATTGCAGTTGACTATAGAATTTGGACATATTGAAAATTCAGTTGTTTATATAAGAAATAT
unknown DNA

TAAATTTTTACACTATTATTTAACATAAAGAGTACTTTGGTAGATAGAATTCAAGGGAGAGTGACATACTTTAGCATAATGATATTTTCA   4770
ATTTAAAAATGTGATAATAAATTGTATTTCTCATGAAACCATCTATCTTAAGTTCCCTCTCACTGTATGAAATCGTATTACTATAAAAGT
unknown DNA
```

```
       PsiI                                 PflMI       SspI                         AseI
AGTGTAATTGTTATAAGAAACAAATACATAAGTTCCATTATTTGGACAAATATTAGACTCTTTCATTGGGTATTAATAATGACCGTAAGT   4860
TCACATTAACAATATTCTTTGTTTATGTATTCAAGGTAATAAACCTGTTTATAATCTGAGAAAGTAACCCATAATTATTACTGGCATTCA
                                             unknown DNA

TCTATTATGTATTTTTCAAAATTCGCTAGTTTGCTTAATTACTCCAAATTGTAAAAAACATTTTGTCTTTTGTGCAAAAAGAAGTGTCGG   4950
AGATAATACATAAAAAGTTTTAAGCGATCAAACGAATTAATGAGGTTTAACATTTTTTGTAAAACAGAAAACACGTTTTTCTTCACAGCC
                                             unknown DNA

                                            MluI        SnaBI                            BsaI DraIII
CCAGATTTTTCATTTTCTGGTGACCAGATAAAGTTCACACACGCGTAGTACGTAATCAGTCAAAACCAAAACCAAATACACTACGTGGAG   5040
GGTCTAAAAAGTAAAAGACCACTGGTCTATTTCAAGTGTGTGCGCATCATGCATTAGTCAGTTTTGGTTTTGGTTTATGTGATGCACCTC
                                             unknown DNA

                 SspI
ACCATGTATAGGCTAATATTAAACTCTAGCTTTACGTCAATCTCATCAANGCAAGAAATAACCTTGATAATCGTCAAATTAGGGACACTT   5130
TGGTACATATCCGATTATAATTTGAGATCGAAATGCAGTTAGAGTAGTTNCGTTCTTTATTGGAACTATTAGCAGTTTAATCCCTGTGAA
                                             unknown DNA

  End (5133)
GTA     3'
            5133
CAT     5'
unknown DNA
```

	Feature	Location	Size (bp)			Type
✓	**AmpR**	1 .. 530	530		←	CDS

2 segments

/product = beta lactamase

/note = beta lactamase

/translation = MSIQHFRVALIPFFAAFCLPVFA,HPETLVKVKDAEDQLGARVGYIELDLNSGKILESFRPEERFPMMSTFKVLLCGAVLSRVDAGQEQLGRRIHYSQNDLVEYSPVTEKHLTDGMTVF
SAAITMSDNTAANLLLTTIGGPKELTAFLPNMGGSCNSALILGKPELNETLPNDE

176 amino acids = 19,3 kDa

	Feature	Location	Size (bp)			Type
✓	**AmpR promoter**	531 .. 635	105		←	promoter

/gene = *bla*

	Feature	Location	Size (bp)			Type
✓	**CaMV 35S promoter**	926 .. 1271	346		→	promoter

/note = strong constitutive promoter from cauliflower mosaic virus

	Feature	Location	Size (bp)			Type
✓	**A1**	1362 .. 2435	1074		→	CDS

/translation = MEGGAGASEKGTVLVTGASGFAGSWLVMKLLQAGYTVRATVRDPANVGKTKPLMDLPGATERLSIWKADLAEEGSFHDAIRGCTGVFHVATPMDFLSKDPENEVIKPTVEGMISIM
CKEAGTVRRIVFTSSAGTVNLEERQRPVYDEESWTDVDFCRRVKMTGWMYFVSKTLAEKAALAYAAEHGLDLVTIIPTLVVGPFISASMPPSLITALALITGNAPHYSILKQVQLIHLDI
DAEIFLFENPAAAGRYVCSSHDVTIHGLAAMLRDRYPEYDVPQRFPGIQDDLQPVRFSSKKLQDLGFTFRYKTLEDMFDAAIRTCQEKGLIPLATAAGGDGFASVRAPGETEATIGA*

357 amino acids = 38,7 kDa

	Feature	Location	Size (bp)			Type
✓	**t35S**	2671 .. 2859	189		⊢⊣	terminator
✓	**lac UV5 promoter**	2901 .. 2931	31		→	promoter

3 segments

/note = *E. coli lac* promoter with an "up" mutation

	Feature	Location	Size (bp)			Type
✓	**lac operator**	2939 .. 2955	17		⊢⊣	protein_bind

/bound_moiety = *lac* repressor encoded by *lacI*

/note = The *lac* repressor binds to the *lac* operator to inhibit transcription in *E. coli*. This inhibition can be relieved by adding lactose or isopropyl-β-D-thiogalactopyranoside (IPTG).

	Feature	Location	Size (bp)			Type
✓	**NOS promoter**	3056 .. 3235	180		→	promoter

/note = nopaline synthase promoter

	Feature	Location	Size (bp)			Type
✓	**NeoR/KanR**	3277 .. 4074	798		→	CDS

/gene = *aph(3')-II* (or *nptII*)

/product = aminoglycoside phosphotransferase from Tn5

/note = confers resistance to neomycin, kanamycin, and G418 (Geneticin®)

/translation = MWIEQDGLHAGSPAAWVERLFGYDWAQQTIGCSDAAVFRLSAQGRPVLFVKTDLSGALNELQDEAARLSWLATTGVPCAAVLDVVTEAGRDWLLLGEVPGQDLLSSHLAPAEKVSII
AMRRLHTLDPATCPFDHQAKHRIERARTRMEAGLVDQDDLDEEHQGLAPAELFARLKARMPDGEDLVVTHGDACLPNIMVENGRFSGFIDCGRLGVADRYQDIALATRDIAEELGC
ADRFLVLYGIAAPDSQRIAFYRLLDEFF*

265 amino acids = 29,2 kDa

	Feature	Location	Size (bp)			Type
✓	**trunc. OCS terminator**	4253 .. 4532	280		⊢⊣	misc_feature

/note = octopine synthase terminator

	Feature	Location	Size (bp)			Type
✓	**unknown DNA**	4533 .. 5133	601		⊢⊣	unsure

DNA Type: Synthetic DNA

Description:

Created: Donnerstag, 16. Nov 2017
Last Modified: Donnerstag, 16. Nov 2017

Accession Number:
Code Number:

Sequence Author:

Comments:

References:
Embedded Files:
