# Supplementary material for: Molecular Characterization and Event-Specific Real-Time PCR Detection of Two Dissimilar Groups of Genetically Modified Petunia (Petunia x hybrida) Sold on the Market
Source: Front Plant Sci. 2020 Jul 14;11:1047. doi: 10.3389/fpls.2020.01047 (PMC7372090; doi:10.3389/fpls.2020.01047)
Supplement: Supplementary file 2 [file DataSheet_2.pdf]

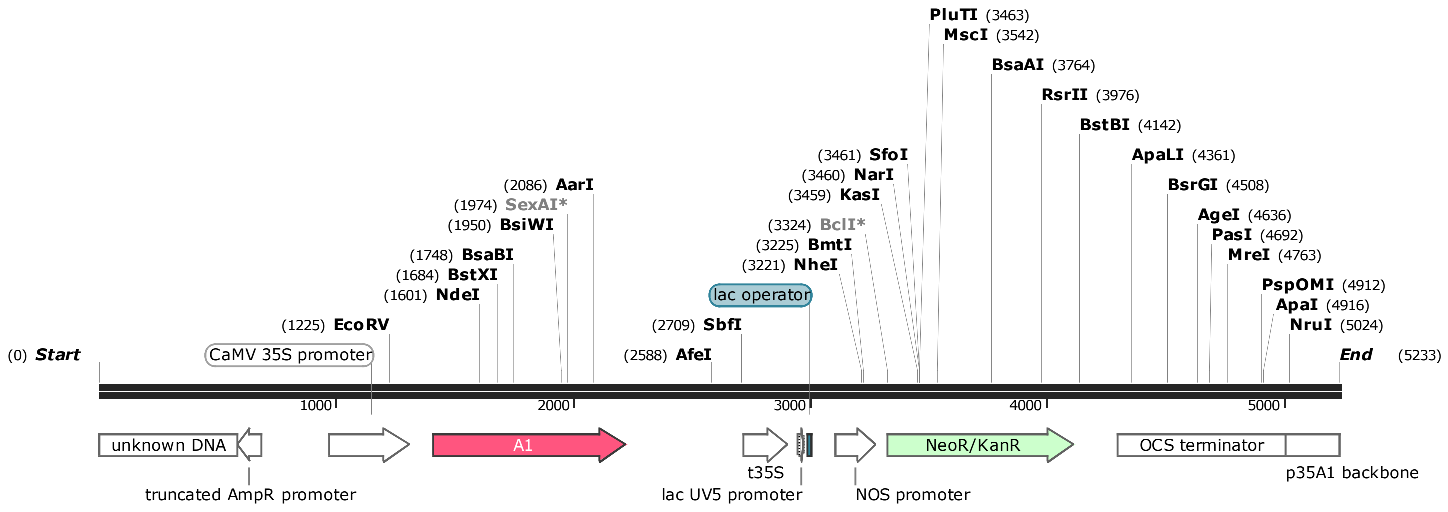

LGL_Contig_pGSMDF-24
5233 bp

Start (0)

5′ CCGAAGCAGTTTCAAGACCTTTCCCTTTTTATTACCTGTAGGGTTTCCTCCATGGATCAGTCATGTGATTTTAGCAACCCATTTGATCCC 90
3′ GGCTTCGTCAAAGTTCTGGAAAGGGAAAAATAATGGACATCCCAAAGGAGGTACCTAGTCAGTACACTAAAATCGTTGGGTAAACTAGGG
unknown DNA

TAACGGTAACAAAAATAAGATATACTTTGAAACCAAGACTCAGTAGAATTTGAAGACTGAAAGTCCTTCACAATCTGATTGGATTTGAAG 180
ATTGCCATTGTTTTTATTCTATATGAAACTTTGGTTCTGAGTCATCTTAAACTTCTGACTTTCAGGAAGTGTTAGACTAACCTAAACTTC
unknown DNA

ACTGCGAAGAGTTTGAAGACTGAAATCTTCTCTATCTGATGTTATGATGCACTATTTTTCTTGTCCCTCGTTAAGTCAAAGTTCAAATAT 270
TGACGCTTCTCAAACTTCTGACTTTAGAAGAGATAGACTACAATACTACGTGATAAAAAGAACAGGGAGCAATTCAGTTTCAAGTTTATA
unknown DNA

AGCAGCACTATAGATATTTAGATGAAAAGTAAAATGTCAGCTTGTATGCTGGATTTTGAGCATGCAAGAAGCAACATAACTTACTACTTA 360
TCGTCGTGATATCTATAAATCTACTTTTCATTTTACAGTCGAACATACGACCTAAAACTCGTACGTTCTTCGTTGTATTGAATGATGAAT
unknown DNA

CAGATATAAACTATGTAGTGTTTAGGATTTTCCTAGATCTTACAGAACAGGAAACTTGATTTTTCACTCTCTGATGAAGTAAGTTGTGAC 450
GTCTATATTTGATACATCACAAATCCTAAAAGGATCTAGAATGTCTTGTCCTTTGAACTAAAAAGTGAGAGACTACTTCATTCAACACTG
unknown DNA

GATGCTATCACCTTCACGGAGGCTCGACAGTGGCATCAAACCTTATTGGCTGTAGTTAATGTTACTTTCTGTTAGGCTTTGCAAAGGAAA 540
CTACGATAGTGGAAGTGCCTCCGAGCTGTCACCGTAGTTTGGAATAACCGACATCAATTACAATGAAAGACAATCCGAAACGTTTCCTTT
unknown DNA

TGTGTTTCCATCTGCATGATCCTACTTTGTGGATTCAGGATGTCCTTTTTCAATATTATTGAAGCATTTATCAGGGTTATTGTCTCATGA 630
ACACAAAGGTAGACGTACTAGGATGAAACACCTAAGTCCTACAGGAAAAAGTTATAATAACTTCGTAAATAGTCCCAATAACAGAGTACT
unknown DNA | truncated AmpR promoter

GCGGATACATATTTGAATGTATTTAAAAAAATAAACAAATAGGGGTTCCGCGCACATTTCCCCGAAAAGTGCCACCTGACGTCTAAGAAA 720
CGCCTATGTATAAACTTACATAAATTTTTTTATTTGTTTATCCCCAAGGCGCGTGTAAAGGGGCTTTTCACGGTGGACTGCAGATTCTTT
truncated AmpR promoter

CCATTATTATCATGACATTAACCTATAAAAATAGGCGTATCACGAGGCCCTTTCGTCTTCAAGAATTCCCATGGAGTCAAAGATTCAAAT 810
GGTAATAATAGTACTGTAATTGGATATTTTTATCCGCATAGTGCTCCGGGAAAGCAGAAGTTCTTAAGGGTACCTCAGTTTCTAAGTTTA

AGAGGACCTAACAGAACTCGCCGTAAAGACTGGCGAACAGTTCATACAGAGTCTCTTACGACTCAATGACAAGAAGAAAATCTTCGTCAA 900
TCTCCTGGATTGTCTTGAGCGGCATTTCTGACCGCTTGTCAAGTATGTCTCAGAGAATGCTGAGTTACTGTTCTTCTTTTAGAAGCAGTT

CATGGTGGAGCACGACACGCTTGTCTACTCCAAAAATATCAAAGATACAGTCTCAGAAGACCAAAGGGCAATTGAGACTTTTCAACAAAG 990
GTACCACCTCGTGCTGTGCGAACAGATGAGGTTTTTATAGTTTCTATGTCAGAGTCTTCTGGTTTCCCGTTAACTCTGAAAAGTTGTTTC
CaMV 35S promoter

GGTAATATCCGGAAACCTCCTCGGATTCCATTGCCCAGCTATCTGTCACTTTATTGTGAAGATAGTGGAAAAGGAAGGTGGCTCCTACAA 1080
CCATTATAGGCCTTTGGAGGAGCCTAAGGTAACGGGTCGATAGACAGTGAAATAACACTTCTATCACCTTTTCCTTCCACCGAGGATGTT
CaMV 35S promoter

ATGCCATCATTGCGATAAAGGAAAGGCCATCGTTGAAGATGCCTCTGCCGACAGTGGTCCCAAAGATGGACCCCCACCCACGAGGAGCAT 1170
TACGGTAGTAACGCTATTTCCTTTCCGGTAGCAACTTCTACGGAGACGGCTGTCACCAGGGTTTCTACCTGGGGGTGGGTGCTCCTCGTA
CaMV 35S promoter

```
                                                               EcoRV
CGTGGAAAAAGAAGACGTTCCAACCACGTCTTCAAAGCAAGTGGATTGATGTGATATCTCCACTGACGTAAGGGATGACGCACAATCCCA   1260
GCACCTTTTTCTTCTGCAAGGTTGGTGCAGAAGTTTCGTTCACCTAACTACACTATAGAGGTGACTGCATTCCCTACTGCGTGTTAGGGT
CaMV 35S promoter

CTATCCTTCGCAAGACCCTTCCTCTATATAAGGAAGTTCATTTCATTTGGAGAGGACAGGGTACCCGGGGATCCTCTAGAGAATTCCAGC   1350
GATAGGAAGCGTTCTGGGAAGGAGATATATTCCTTCAAGTAAAGTAAACCTCTCCTGTCCCATGGGCCCCTAGGAGATCTCTTAAGGTCG
CaMV 35S promoter

TGCTCACTCAGTCCTGCGCAAGAGCTCGCTCTCGGAGAAAAAAACGCGGGAGGCGATAATGGAGGGAGGTGCCGGTGCGAGCGAGAAAGG   1440
ACGAGTGAGTCAGGACGCGTTCTCGAGCGAGAGCCTCTTTTTTTGCGCCCTCCGCTATTACCTCCCTCCACGGCCACGCTCGCTCTTTCC
1 5 10
M E G G A G A S E K G
A1

GACGGTGCTGGTCACGGGGGCGTCGGGCTTCGCCGGCTCCTGGCTCGTCATGAAGCTCCTCCAGGCCGGCTACACCGTCCGGGCGACCGT   1530
CTGCCACGACCAGTGCCCCCGCAGCCCGAAGCGGCCGAGGACCGAGCAGTACTTCGAGGAGGTCCGGCCGATGTGGCAGGCCCGCTGGCA
15 20 25 30 35 40
T V L V T G A S G F A G S W L V M K L L Q A G Y T V R A T V
A1

                                                                      NdeI
GCGCGATCCCGCGAACGTTGGGAAGACGAAGCCATTGATGGACCTTCCCGGAGCAACGGAGCGCCTGTCCATATGGAAAGCCGACCTGGC   1620
CGCGCTAGGGCGCTTGCAACCCTTCTGCTTCGGTAACTACCTGGAAGGGCCTCGTTGCCTCGCGGACAGGTATACCTTTCGGCTGGACCG
45 50 55 60 65 70
R D P A N V G K T K P L M D L P G A T E R L S I W K A D L A
A1

                                                               BstXI
GGAGGAAGGCAGCTTCCACGACGCCATCAGGGGCTGCACCGGCGTCTTCCACGTCGCCACGCCCATGGACTTCCTGTCCAAAGACCCTGA   1710
CCTCCTTCCGTCGAAGGTGCTGCGGTAGTCCCCGACGTGGCCGCAGAAGGTGCAGCGGTGCGGGTACCTGAAGGACAGGTTTCTGGGACT
75 80 85 90 95 100
E E G S F H D A I R G C T G V F H V A T P M D F L S K D P E
A1

                                      BsaBI
GAATGAGGTAATCAAGCCGACGGTGGAAGGGATGATAAGCATCATGCGGGCATGCAAGGAGGCCGGCACCGTGCGGCGCATCGTCTTCAC   1800
CTTACTCCATTAGTTCGGCTGCCACCTTCCCTACTATTCGTAGTACGCCCGTACGTTCCTCCGGCCGTGGCACGCCGCGTAGCAGAAGTG
105 110 115 120 125 130
N E V I K P T V E G M I S I M R A C K E A G T V R R I V F T
A1

TTCCTCCGCCGGGACGGTCAACCTGGAGGAGCGGCAGAGGCCCGTCTACGACGAGGAAAGCTGGACCGACGTCGACTTCTGCCGTCGCGT   1890
AAGGAGGCGGCCCTGCCAGTTGGACCTCCTCGCCGTCTCCGGGCAGATGCTGCTCCTTTCGACCTGGCTGCAGCTGAAGACGGCAGCGCA
135 140 145 150 155 160
S S A G T V N L E E R Q R P V Y D E E S W T D V D F C R R V
A1

                                                              BsiWI                 SexAI*
CAAGATGACAGGATGGATGTACTTCGTGTCTAAAACCCTGGCGGAGAAGGCGGCCCTGGCGTACGCGGCGGAGCACGGCCTGGACCTGGT   1980
GTTCTACTGTCCTACCTACATGAAGCACAGATTTTGGGACCGCCTCTTCCGCCGGGACCGCATGCGCCGCCTCGTGCCGGACCTGGACCA
165 170 175 180 185 190
K M T G W M Y F V S K T L A E K A A L A Y A A E H G L D L V
A1

CACCATCATCCCGACGCTCGTGGTCGGCCCGTTCATCAGCGCGTCCATGCCGCCCAGCCTCATCACCGCGCTGGCGCTCATCACGGGGAA   2070
GTGGTAGTAGGGCTGCGAGCACCAGCCGGGCAAGTAGTCGCGCAGGTACGGCGGGTCGGAGTAGTGGCGCGACCGCGAGTAGTGCCCCTT
195 200 205 210 215 220
T I I P T L V V G P F I S A S M P P S L I T A L A L I T G N
A1
```

AarI

```
CGCGCCGCACTACTCGATCCTCAAGCAGGTGCAGCTCATCCACCTCGACGACCTCTGCGACGCCGAGATCTTCCTCTTCGAGAACCCGGC  2160
GCGCGGCGTGATGAGCTAGGAGTTCGTCCACGTCGAGTAGGTGGAGCTGCTGGAGACGCTGCGGCTCTAGAAGGAGAAGCTCTTGGGCCG
        225        230        235        240        245        250
 A  P  H  Y  S  I  L  K  Q  V  Q  L  I  H  L  D  D  L  C  D  A  E  I  F  L  F  E  N  P  A
```
A1

```
CGCGGCCGGGCGCTACGTTTGCTCCTCGCACGACGTCACCATCCACGGCCTCGCCGCCATGCTCcAGGGATAGGTACCCCGaAGTACGAC  2250
GCGCCGGCCCGCGATGCAAACGAGGAGCGTGCTGCAGTGGTAGGTGCCGGAGCGGCGGTACGAGgTCCCTATCCATGGGGCtTCATGCTG
        255        260        265        270        275
 A  A  G  R  Y  V  C  S  S  H  D  V  T  I  H  G  L  A  A  M  L  Q  G  *
```
A1

```
GTCCCGCARAGGTTCCCCGGGATCCAGGACGACCTCCAGCCGGTGCGCTTCTCGTCCAAGAAGCTCCAGGACCTCGGGTTCACCTTCAGG  2340
CAGGGCGTYTCCAAGGGGCCCTAGGTCCTGCTGGAGGTCGGCCACGCGAAGAGCAGGTTCTTCGAGGTCCTGGAGCCCAAGTGGAAGTCC

TACAAGACGCTGGAGGACATGTTCGACGCCGCCATCCGGACTTGCCAGGAGAAGGGCCTCATCCCCCTCGCCACTGCCGCCGGAGGGGAC  2430
ATGTTCTGCGACCTCCTGTACAAGCTGCGGCGGTAGGCCTGAACGGTCCTCTTCCCGGAGTAGGGGGAGCGGTGACGGCGGCCTCCCCTG

GGCTTTGCCTCGGTGCGCGCACCCGGCGAGACGGAGGCGACGATTGGCGCTTAGGCAACGATCCCCCGGCTYTCCCCGTYGATATGATGC  2520
CCGAAACGGAGCCACGCGCGTGGGCCGCTCTGCCTCCGCTGCTAACCGCGAATCCGTTGCTAGGGGGCCGARAGGGGCARCTATACTACG
```

AfeI

```
AATCAGCTATCTATYTCTTGTTTGCCAAAAAAAAaTAAgGGGAGGTCTTGGCATACTCGATCTAGAGCGCTTTGCAAGAGCGTTAAGGCT  2610
TTAGTCGATAGATARAGAACAAACGGTTTTTTTTtATTcCCCTCCAGAACCGTATGAGCTAGATCTCGCGAAACGTTCTCGCAATTCCGA

TAGATGACTATGGCTACGATGGACGAATAGAGACAAAGCATGGACTGGGTTGCAATTAAAAAAAAAAAAAAAAAAGGAATTCTCTAGAGT  2700
ATCTACTGATACCGATGCTACCTGCTTATCTCTGTTTCGTACCTGACCCAACGTTAATTTTTTTTTTTTTTTTTTCCTTAAGAGATCTCA
```

SbfI

```
CGACCTGCAGGCATGCCCGCTGAAATCACCAGTCTCTCTCTACAAATCTATCTCTCTCTATAATAATGTGTGAGTAGTTCCCAGATAAGG  2790
GCTGGACGTCCGTACGGGCGACTTTAGTGGTCAGAGAGAGATGTTTAGATAGAGAGAGATATTATTACACACTCATCAAGGGTCTATTCC
```
t35S

```
GAATTAGGGTTCTTATAGGGTTTCGCTCATGTGTTGAGCATATAAGAAACCCTTAGTATGTATTTGTATTTGTAAAATACTTCTATCAAT  2880
CTTAATCCCAAGAATATCCCAAAGCGAGTACACAACTCGTATATTCTTTGGGAATCATACATAAACATAAACATTTTATGAAGATAGTTA
```
t35S

```
AAAATTTCTAATTCCTAAAACCAAAATCCAGGGGTACCGAGCTCGAATTCTCACTCATTAGGCACCCCAGGCTTTACACTTTATGCTTCC  2970
TTTTAAAGATTAAGGATTTTGGTTTTAGGTCCCCATGGCTCGAGCTTAAGAGTGAGTAATCCGTGGGGTCCGAAATGTGAAATACGAAGG
```
t35S

-35

lac UV5 promoter

```
GGCTCGTATAATGTGTGGAATTGTGAGCGGATAACAATTTCACACAGGAAACAGGATCATGAGCGGAGAATTAAGGGAGTCACGTTATGA  3060
CCGAGCATATTACACACCTTAACACTCGCCTATTGTTAAAGTGTGTCCTTTGTCCTAGTACTCGCCTCTTAATTCCCTCAGTGCAATACT
```
-10

lac UV5 promoter

lac operator

```
CCCCCGCCGATGACGCGGGACAAGCCGTTTTACGTTTGGAACTGACAGAACCGCAACGTTGAAGGAGCCACTCAGCCGCGGGTTTCTGGA  3150
GGGGGCGGCTACTGCGCCCTGTTCGGCAAAATGCAAACCTTGACTGTCTTGGCGTTGCAACTTCCTCGGTGAGTCGGCGCCCAAAGACCT
```
NOS promoter

NheI BmtI

```
GTTTAATGAGCTAAGCACATACGTCAGAAACCATTATTGCGCGTTCAAAAGTCGCCTAAGGTCACTATCAGCTAGCAAATATTTCTTGTC  3240
CAAATTACTCGATTCGTGTATGCAGTCTTTGGTAATAACGCGCAAGTTTTCAGCGGATTCCAGTGATAGTCGATCGTTTATAAAGAACAG
```
NOS promoter

BclI*

AAAAATGCTCCACTGACGTTCCATAAATTCCCCTCGGTATCCAATTAGAGTCTCATATTCACTCTCAATCCAGATCCGGCCCATGATCAT 3330
TTTTTACGAGGTGACTGCAAGGTATTTAAGGGGAGCCATAGGTTAATCTCAGAGTATAAGTGAGAGTTAGGTCTAGGCCGGGTACTAGTA

NOS promoter
1 M
NeoR/KanR

GTGGATTGAACAAGATGGATTGCACGCAGGTTCTCCGGCCGCTTGGGTGGAGAGGCTATTCGGCTATGACTGGGCACAACAGACAATCGG 3420
CACCTAACTTGTTCTACCTAACGTGCGTCCAAGAGGCCGGCGAACCCACCTCTCCGATAAGCCGATACTGACCCGTGTTGTCTGTTAGCC

5 10 15 20 25 30
W I E Q D G L H A G S P A A W V E R L F G Y D W A Q Q T I G
NeoR/KanR

KasI NarI SfoI PluTI

CTGCTCTGATGCCGCCGTGTTCCGGCTGTCAGCGCAGGGGCGCCCGGTTCTTTTTGTCAAGACCGACCTGTCCGGTGCCCTGAATGAACT 3510
GACGAGACTACGGCGGCACAAGGCCGACAGTCGCGTCCCCGCGGGCCAAGAAAAACAGTTCTGGCTGGACAGGCCACGGGACTTACTTGA

35 40 45 50 55 60
C S D A A V F R L S A Q G R P V L F V K T D L S G A L N E L
NeoR/KanR

MscI

GCAGGACGAGGCAGCGCGGCTATCGTGGCTGGCCACGACGGGCGTTCCTTGCGCAGCTGTGCTCGACGTTGTCACTGAAGCGGGAAGGGA 3600
CGTCCTGCTCCGTCGCGCCGATAGCACCGACCGGTGCTGCCCGCAAGGAACGCGTCGACACGAGCTGCAACAGTGACTTCGCCCTTCCCT

65 70 75 80 85 90
Q D E A A R L S W L A T T G V P C A A V L D V V T E A G R D
NeoR/KanR

CTGGCTGCTATTGGGCGAAGTGCCGGGGCAGGATCTCCTGTCATCTCACCTTGCTCCTGCCGAGAAAGTATCCATCATGGCTGATGCAAT 3690
GACCGACGATAACCCGCTTCACGGCCCCGTCCTAGAGGACAGTAGAGTGGAACGAGGACGGCTCTTTCATAGGTAGTACCGACTACGTTA

95 100 105 110 115 120
W L L G E V P G Q D L L S S H L A P A E K V S I M A D A M
NeoR/KanR

BsaAI

GCGGCGGCTGCATACGCTTGATCCGGCTACCTGCCCATTCGACCACCAAGCGAAACATCGCATCGAGCGAGCACGTACTCGGATGGAAGC 3780
CGCCGCCGACGTATGCGAACTAGGCCGATGGACGGGTAAGCTGGTGGTTCGCTTTGTAGCGTAGCTCGCTCGTGCATGAGCCTACCTTCG

125 130 135 140 145 150
R R L H T L D P A T C P F D H Q A K H R I E R A R T R M E A
NeoR/KanR

CGGTCTTGTCGATCAGGATGATCTGGACGAAGAGCATCAGGGGCTCGCGCCAGCCGAACTGTTCGCCAGGCTCAAGGCGCGCATGCCCGA 3870
GCCAGAACAGCTAGTCCTACTAGACCTGCTTCTCGTAGTCCCCGAGCGCGGTCGGCTTGACAAGCGGTCCGAGTTCCGCGCGTACGGGCT

155 160 165 170 175 180
G L V D Q D D L D E E H Q G L A P A E L F A R L K A R M P D
NeoR/KanR

CGGCGAGGATCTCGTCGTGACCCATGGCGATGCCTGCTTGCCGAATATCATGGTGGAAAATGGCCGCTTTTCTGGATTCATCGACTGTGG 3960
GCCGCTCCTAGAGCAGCACTGGGTACCGCTACGGACGAACGGCTTATAGTACCACCTTTTACCGGCGAAAAGACCTAAGTAGCTGACACC

185 190 195 200 205 210
G E D L V V T H G D A C L P N I M V E N G R F S G F I D C G
NeoR/KanR

RsrII

CCGGCTGGGTGTGGCGGACCGCTATCAGGACATAGCGTTGGCTACCCGTGATATTGCTGAAGAGCTTGGCGGCGAATGGGCTGACCGCTT 4050
GGCCGACCCACACCGCCTGGCGATAGTCCTGTATCGCAACCGATGGGCACTATAACGACTTCTCGAACCGCCGCTTACCCGACTGGCGAA

215 220 225 230 235 240
R L G V A D R Y Q D I A L A T R D I A E E L G G E W A D R F
NeoR/KanR

```
CCTCGTGCTTTACGGTATCGCCGCTCCCGATTCGCAGCGCATCGCCTTCTATCGCCTTCTTGACGAGTTCTTCTGAGCGGGACTCTGGGG   4140
GGAGCACGAAATGCCATAGCGGCGAGGGCTAAGCGTCGCGTAGCGGAAGATAGCGGAAGAACTGCTCAAGAAGACTCGCCCTGAGACCCC
       245            250            255            260            265
 L  V  L  Y  G  I  A  A  P  D  S  Q  R  I  A  F  Y  R  L  L  D  E  F  F  *
NeoR/KanR

 BstBI
TTCGAAATGACCGACCAAGCGACGCCCAACCTGCCATCACGARaATTTCGATTCCACCGCCGCCTTCWATGAAAGGTTGGGCTTCGGAAT   4230
AAGCTTTACTGGCTGGTTCGCTGCGGGTTGGACGGTAGTGCTYtTAAAGCTAAGGTGGCGGCGGAAGWTACTTTCCAACCCGAAGCCTTA

CGTTTTCCGGGACGCCGGCTGGATGATCCTCCAGCGCGGGGATCTCATGCTGGAGTTCTTCGCCCACCCCCTGCTTTAATGAGATATGCG   4320
GCAAAAGGCCCTGCGGCCGACCTACTAGGAGGTCGCGCCCCTAGAGTACGACCTCAAGAAGCGGGTGGGGGACGAAATTACTCTATACGC
                                                                              OCS terminator

                                          ApaLI
AGACGCCTATGATCGCATGATATTTGCTTTCAATTCTGTTGTGCACGTTGTAAAAAACCTGAGCATGTGTAGCTCAGATCCTTACCGCCG   4410
TCTGCGGATACTAGCGTACTATAAACGAAAGTTAAGACAACACGTGCAACATTTTTTGGACTCGTACACATCGAGTCTAGGAATGGCGGC
OCS terminator

GTTTCGGTTCATTCTAATGAATATATCACCCGTTACTATCGTATTTTTATGAATAATATTCTCCGTTCAATTTACTGATTGTACCCTACT   4500
CAAAGCCAAGTAAGATTACTTATATAGTGGGCAATGATAGCATAAAAATACTTATTATAAGAGGCAAGTTAAATGACTAACATGGGATGA
OCS terminator

       BsrGI
ACTTATATGTACAATATTAAAATGAAAACAATATATTGTGCTGAATAGGTTTATAGCGACATCTATGATAGAGCGCCACAATAACAAACA   4590
TGAATATACATGTTATAATTTTACTTTTGTTATATAACACGACTTATCCAAATATCGCTGTAGATACTATCTCGCGGTGTTATTGTTTGT
OCS terminator

                                               AgeI
ATTGCGTTTTATTATTACAAATCCAATTTTAAAAAAAGCGGCAGAACCGGTCAAACCTAAAAGACTGATTACATAAATCTTATTCAAATT   4680
TAACGCAAAATAATAATGTTTAGGTTAAAATTTTTTTCGCCGTCTTGGCCAGTTTGGATTTTCTGACTAATGTATTTAGAATAAGTTTAA
OCS terminator

           PasI                                                                      MreI
TCAAAAGTGCCCCAGGGGCTAGTATCTACGACACACCGAGCGGCGAACTAATAACGCTCACTGAAGGGAACTCCGGTTCCCCGCCGGCGC   4770
AGTTTTCACGGGGTCCCCGATCATAGATGCTGTGTGGCTCGCCGCTTGATTATTGCGAGTGACTTCCCTTGAGGCCAAGGGGCGGCCGCG
OCS terminator

GCATGGGTGAGATTCCTTGAAGTTGAGTATTGGCCGTCCGCTCTACCGAAAGTTACGGGCACCATTCAACCCGGTCCAGCACGGCGGCCG   4860
CGTACCCACTCTAAGGAACTTCAACTCATAACCGGCAGGCGAGATGGCTTTCAATGCCCGTGGTAAGTTGGGCCAGGTCGTGCCGCCGGC
OCS terminator

                                                  PspOMI ApaI
GGTAACCGACTTGCTGCCCCGAGAATTATGCAGCATTTTTTTGGTGTATGTGGGCCCCAAATGAAGTGCAGGTCAAACCTTGACAGTGAC   4950
CCATTGGCTGAACGACGGGGCTCTTAATACGTCGTAAAAAAACCACATACACCCGGGGTTTACTTCACGTCCAGTTTGGAACTGTCACTG
OCS terminator

                                                                            NruI
GACAAATCGTTGGGCGGGTCCAGGGCGAATTTTGCGACAACATGTCGAGGCTCAGCAGGGGCTCGATCCCCTCGCGAGTTGGTTCAGCTG   5040
CTGTTTAGCAACCCGCCCAGGTCCCGCTTAAAACGCTGTTGTACAGCTCCGAGTCGTCCCCGAGCTAGGGGAGCGCTCAACCAAGTCGAC
OCS terminator                                              p35A1 backbone

CTGCCTGAGGCTGGACGACCTCGCGGAGTTCTACCGGCAGTGCAAATCCGTCGGCATCCAGGAAACCAGCAGCGGCTATCCGCGCATCCA   5130
GACGGACTCCGACCTGCTGGAGCGCCTCAAGATGGCCGTCACGTTTAGGCAGCCGTAGGTCCTTTGGTCGTCGCCGATAGGCGCGTAGGT
p35A1 backbone
```

```
TGCCCCCGAACTGCCAGGAATGGGGAAGGCACAATGGCCCCCTTTGGTCCATCCAACCGATGCCCTTGAAAACCTTCAACCCAGTCAGCT
                                                                                                   5220
ACGGGGGCTTGACGGTCCTTACCCCTTCCGTGTTACCGGGGGAAACCAGGTAGGTTGGCTACGGGAACTTTTGGAAGTTGGGTCAGTCGA
                                   p35A1 backbone

              End (5233)
CCTTCCGGTGGGC     3'
                     5233
GGAAGGCCACCCG     5'
  p35A1 backbone
```

DNA Type: Synthetic DNA

Description:

Created: Donnerstag, 16. Nov 2017
Last Modified: Donnerstag, 16. Nov 2017

Accession Number:
Code Number:

Sequence Author:

Comments:

References:
Embedded Files:
