# Supplementary material for: Molecular Characterization and Event-Specific Real-Time PCR Detection of Two Dissimilar Groups of Genetically Modified Petunia (Petunia x hybrida) Sold on the Market
Source: Front Plant Sci. 2020 Jul 14;11:1047. doi: 10.3389/fpls.2020.01047 (PMC7372090; doi:10.3389/fpls.2020.01047)
Supplement: Supplementary file 3 [file DataSheet_3.pdf]

G1

Clusters based on MinION data

```
Cluster_11.mafft_consensus      ------------------------------------------------------------
Cluster_13.mafft_consensus      ------------------------------------------------------------
Cluster_8.mafft_consensus       --------------GCGCTGTACAGTGTATGTTGTGTAAATATTGAACTCCCACAGAGAT
Cluster_7.mafft_consensus       ------------------------------------------------------------
Cluster_9.mafft_consensus       ------------------------------------------------------------
Cluster_3.mafft_consensus       GACTAGTCGGGACCAGTACCGGTAAGAAATGATCCAGTTGTCGTTGTTTATGGTCTGGTT
Cluster_5.mafft_consensus       ------------------------------------------------------------
Cluster_2.mafft_consensus       -------------GGAGATGGATGACTTATGACACAGAGATTCCAGACTATTTAAAAGAT
Cluster_6.mafft_consensus       ------------------------------------------------------------
Cluster_1.mafft_consensus       ------------------------------------------------------------
Cluster_4.mafft_consensus       ------------------------------------------------------------


Cluster_11.mafft_consensus      ------------------------------------------------------------
Cluster_13.mafft_consensus      ------------------------------------------------------------
Cluster_8.mafft_consensus       TCAAAGGCAGAGAATTTGAAAAATTAAATGAAAGATCTTGAAAGACAAATTTTGTTCTTC
Cluster_7.mafft_consensus       ------------------------------------------------------------
Cluster_9.mafft_consensus       ------------------------------------------------------------
Cluster_3.mafft_consensus       TATAATTGCTGTGTGATGACTGAATATTATGACAGATCAAGAATTTGAAGAAATTATAAA
Cluster_5.mafft_consensus       ------------------------------------------------------------
Cluster_2.mafft_consensus       TTGAAATGAAAGATCTTGAAAATTTGTTTGG-----------------------------
Cluster_6.mafft_consensus       ------------------------------------------------------------
Cluster_1.mafft_consensus       ------------------------------------------------------------
Cluster_4.mafft_consensus       ------------------------------------------------------------


Cluster_11.mafft_consensus      ------------------------------------------------------------
Cluster_13.mafft_consensus      ------------------------------------------------------------
Cluster_8.mafft_consensus       AAATTGAGCATTTGATAAATGGAATTTTTATCCATCAATCAACATACACTGAAAAGATTT
Cluster_7.mafft_consensus       ------------------------------------------------------------
Cluster_9.mafft_consensus       ------------------------------------------------------------
Cluster_3.mafft_consensus       TTCTGTAGGCATTTGATAAATGG--TTTTATCCATCAATCAACATACACTGAAAAGATTT
Cluster_5.mafft_consensus       ------------------------------------------------------------
Cluster_2.mafft_consensus       ---TTCAATCATTTGATAAATGGAATTTTATCCATCAATCAACATACACTGAAAAGATTT
Cluster_6.mafft_consensus       ------------------------------------------------------------
Cluster_1.mafft_consensus       ------------------------------------------------------------
Cluster_4.mafft_consensus       ------------------------------------------------------------


Cluster_11.mafft_consensus      --------------------------CGACACGCGGCTGGCCGATGGTCGTGAGA----G
Cluster_13.mafft_consensus      ------------------------------------------------------------
Cluster_8.mafft_consensus       AAAGAGATTTTATATGGATAAA---TCATCCATTGAGTAATCGATGGT--GGAGATCGCT
Cluster_7.mafft_consensus       ------------------------------------------------------------
Cluster_9.mafft_consensus       ------------------------------------------------------------
Cluster_3.mafft_consensus       TAAAGAGATTTTTATGGATAAATATCCATTGAGTA-------GATGGTCGTGAGATCGCT
Cluster_5.mafft_consensus       ------------------------------------------------------------
Cluster_2.mafft_consensus       TAAAGAGATTTTATATGGATAAATCTCATCCATTGAGTATCCGATGGTCGTGAGATCGCT
Cluster_6.mafft_consensus       ------------------------------------------------------------
Cluster_1.mafft_consensus       ------------------------------------------------------------
Cluster_4.mafft_consensus       ------------------------------------------------------------


Cluster_11.mafft_consensus      TGACATAAATAAAGATCCATTTCGCCCTCATGAAAATGATGAAAGATTCTTGGTGCTGAA
Cluster_13.mafft_consensus      ------------------------------------------------------------
Cluster_8.mafft_consensus       TGACATAAATAAAGATCCATTTC-CCCTCATGAAAATGATGAAAGATTCTTGGTGCTGAA
Cluster_7.mafft_consensus       ------------------------------------------------------------
Cluster_9.mafft_consensus       ------------------------------------------------------------
Cluster_3.mafft_consensus       TGACATAAATAAAGATCCATTTC-CCCTCATGAAAATGATGAAA----TTTGGTGCTGAA
Cluster_5.mafft_consensus       ------------------------------------------------------------
Cluster_2.mafft_consensus       TGACATAAATAAAGATCCATTTC-CCCTCATGAAAATGATGAAAGATTCTTGGTGCTGAA
Cluster_6.mafft_consensus       ------------------------------------------------------------
Cluster_1.mafft_consensus       ------------------------------------------------------------
Cluster_4.mafft_consensus       ------------------------------------------------------------


Cluster_11.mafft_consensus      AAACCATATCTTAGTGCA--------TATAATGTATCTTGCTAATAATAC-------GAC
Cluster_13.mafft_consensus      ------------------------------------------------------------
Cluster_8.mafft_consensus       ACACCATATCTTAGTGCAATTGGG--ATTAATG------ATGCAATAAACCCGACCAGAC
Cluster_7.mafft_consensus       ------------------------------------------------------------
Cluster_9.mafft_consensus       ------------------------------------------------------------
Cluster_3.mafft_consensus       ACACCATATCTTAGTGCAATTGGGG-ATTAATGTATCTTGCTAATAATACCCGACCAGAC
Cluster_5.mafft_consensus       ------------------------------------------------------------
```

```
Cluster_2.mafft_consensus       ACACCATATCTTAGTGCAATTGGGGCATTAATGTATCTTGCTAATAATACCCGACCAGAC
Cluster_6.mafft_consensus       ------------------------------------------------------------
Cluster_1.mafft_consensus       -----------------AGTACGGGGATTAATGTATCTTGC----AATAAACGACCAGAC
Cluster_4.mafft_consensus       ------------------------------------------------------------


Cluster_11.mafft_consensus      ATTGC-TTTTCTGTAAACTTATTAGCAAGATTTAGTTCTTCCC--ACACAAAGACACTGG
Cluster_13.mafft_consensus      ----------------------------------------------CACA----------
Cluster_8.mafft_consensus       ATTGC-TTTTCTGTAAACTTATTAGCAAGATTTAGTTC-TCCCCGACACAAAGACACTGG
Cluster_7.mafft_consensus       ------------------------------------------------------------
Cluster_9.mafft_consensus       ------------------------------------------------------------
Cluster_3.mafft_consensus       ATTGC-TTTTCTGTAAACTTATTAGCAAGATTTAGTTCTTCCTCGACACAAAGACACTGG
Cluster_5.mafft_consensus       ------------------------------------------------------------
Cluster_2.mafft_consensus       ATTGCTTTTTCTGTAAACTTATTAGCAAGATTTAGTTCTTCCCCGACACAAAGACACTGG
Cluster_6.mafft_consensus       ------------------------------------------------------------
Cluster_1.mafft_consensus       ATTGCTTTTTCTGTAAACTTATTAGCAAGATTTAGTTCTTCCCCGACACAAAGACACTGG
Cluster_4.mafft_consensus       ------------------------------------------------------------


Cluster_11.mafft_consensus      AATGGTATCAAACATTTATTCAGATACCTCTGAGGAACAATCGACAAA-GTTTGTTTTGC
Cluster_13.mafft_consensus      ------------------------------------------------------------
Cluster_8.mafft_consensus       AATGGTATCAAACATTTATTCAGATACCTCCGAGGAACAATCGACAAAGGTTTGTTTTAT
Cluster_7.mafft_consensus       ------------------------------------------------------------
Cluster_9.mafft_consensus       ------------------------------------------------------------
Cluster_3.mafft_consensus       AATGGTATCAAACATTTATTCAGATACCTCCGAGGAACAATCGACAAAGGTTTGTTTTAT
Cluster_5.mafft_consensus       ------------------------------------------------------------
Cluster_2.mafft_consensus       AATGGTATCAAACATTTATTCAGATACCTCCGAGGAACAATCGACAAAGGTTTGTTTTAT
Cluster_6.mafft_consensus       --------CGCACAATTGT-----------------------------------------
Cluster_1.mafft_consensus       AATGGTATCAAACATTTATTCAGATACCTCCGAGGAACAATCGACAAAGGTTTGTTTTAT
Cluster_4.mafft_consensus       ------------------------------------------------------------


Cluster_11.mafft_consensus      CCAAGTGAATCCAACTTGCCACTAATTGGTTATGCAGAAGTAAGTTGGCCGCAGTG-TTA
Cluster_13.mafft_consensus      ------------------------------------------------CTGTGGTG--TA
Cluster_8.mafft_consensus       TCAAGTGAATCCAACTTGCCACCAATTGGTTATGCAGAAGTAAGTTGGCCGCAGTG-TTA
Cluster_7.mafft_consensus       ---------------------------------GCTAGTACGGGATTGCCGTGGCGGTTA
Cluster_9.mafft_consensus       ----------------------------------------TCAGGGCGCCGC-------A
Cluster_3.mafft_consensus       TCAAGTGAATCCAACTTGCCACCAATTGGTTATGCAGAAGTAAGTTGGCCGCAGTG-TTA
Cluster_5.mafft_consensus       ---------------------------------------ATGTGTTGGCCGCAGTG--TA
Cluster_2.mafft_consensus       TCAAGTGAATCCAACTTGCCACTAATTGGTTATGCAGAAGTAAGTTGGCCGCAGTG-TTA
Cluster_6.mafft_consensus       ------------------------------------------AGGTAGGCGCAGTG-TTA
Cluster_1.mafft_consensus       TCAAGTGAATCCAACTTGCCACCAATTGGTTATGCAGAAGTAAGTTGGCCGCAGTG-TTA
Cluster_4.mafft_consensus       -----------------------------------------------------------A
                                                                                           *

Cluster_11.mafft_consensus      TCACTCATGGTTA---TGGCAGCACTGCATAATCTT--TTACTGTCATGCCATCCGTAAG
Cluster_13.mafft_consensus      TCACTTTGAG----------------GCATAATTCTC-TTACTGTCATGTCATCCGTAAG
Cluster_8.mafft_consensus       TCACTCATGGTTA---TGGCAGCACTGCATAATTCTCCTTACTGTCATGCCATCCGTAAG
Cluster_7.mafft_consensus       TCAC-CATGGTTA---TGGCAGCACTGCATAATTCTT-TTACTGTCATGCCATCCGTAAG
Cluster_9.mafft_consensus       TTACCCATGGTTAGAGTGGCAGCACTGCATAACCTT--TTACTGTCATGCCATCCGTAAG
Cluster_3.mafft_consensus       TCACTCATGGTTA---TGGCAGCACTGCATAATTCTC-TTACTGTCATGCCATCCGTAAG
Cluster_5.mafft_consensus       TCAC-CATGGTTA---TGGCAGCACTGCATAA-TTTCTTTACTGTCATGCCATCCGTAAG
Cluster_2.mafft_consensus       TCACTCATGGTTA---TGGCAGCACTGCATAATTCTCTTTACTGTCATGCCATCCGTAAG
Cluster_6.mafft_consensus       TCACTCATGGTTA---TGGCAGCACTGCATAATTCTCTTTACTGTCATGCCATCCGTAAG
Cluster_1.mafft_consensus       TCACTCATGGTTA---TGGCAGCACTGCATAATTTTT-TTACTGTCATGCCATCCGTAAG
Cluster_4.mafft_consensus       TCC------GGTA---TGGCAGCACTGCATAATTTC--TTACTGTCATGCCATCCGTAAG
                                *        *                ******      *********** **********

Cluster_11.mafft_consensus      ATGCTTTTCTGTGACTGGTGAGTACTCAACCAAGCCATTCTGGAGAATAGTGTATGCGGC
Cluster_13.mafft_consensus      ATGCTTTTCTGTGACTGGTGAGTACTCAACCAAGTCATTCT-GAGAATAGTGTATGCGGC
Cluster_8.mafft_consensus       ATGCTTTTCTGTGACTGGTGAGTACTCAACCAAGTCATTCT-GAGAATAGTGTATGCGGC
Cluster_7.mafft_consensus       ATGCTTTTCTGTGACTGGTGAGTACTCAACCAAGTCATTCT-GAGAATAGTGTATGCGGC
Cluster_9.mafft_consensus       ATGCTTTTCTGTGACTGGTGAGTACTCAACCAAGTCATTCT-GAGAATAGTGTATGCGGC
Cluster_3.mafft_consensus       ATGCTTTTCTGTGACTGGTGAGTACTCAACCAAGTCATTCT-GAGAATAGTGTATGCGGC
Cluster_5.mafft_consensus       ATGCTTTTCTGTGACTGGTGAGTACTCAACCAAGTCATTCT-GAGAATAGTGTATGCGGC
Cluster_2.mafft_consensus       ATGCTTTTCTGTGACTGGTGAGTACTCAACCAAGTCATTCTGGAGAATAGTGTATGCGGC
Cluster_6.mafft_consensus       ATGCTTTTCTGTGACTGGTGAGTACTCAACCAAGTCATTCT-GAGAATAGTGTATGCGGC
Cluster_1.mafft_consensus       ATGCTTTTCTGTGACTGGTGAGTACTCAACCAAGTCATTCT-GAGAATAGTGTATGCGGC
Cluster_4.mafft_consensus       ATGCTTTTCTGTGACTGGTGAGTACTCAACCAAGTCATTCT-GAGAATAGTGTATGCGGC
                                ********************************** ****** ******************

Cluster_11.mafft_consensus      GACCGAGTTGCTCTTGCCCGGCGCTCAACACGGGATAATACCGCGCCACATAGCAGAAC-
Cluster_13.mafft_consensus      GACCGAGTTGTTTTGCCCCGGCG-TCAACACGGGATAATACCGCGCCACATAGCAGAAC-
Cluster_8.mafft_consensus       GACCGAGTTGCTCTTGCCCGGCG-TCAACACGGGATAATACCGCGCCACATAGCAGAAC-
Cluster_7.mafft_consensus       GACCGAGTTGCTCTTGCCCGGCG-TCAACACGGGATAATACCGCGCCACATAGCAGAAC-
Cluster_9.mafft_consensus       GACCGAGTTGCTCTTGCCCGGCG-TCAACACGGGATAATACCGCGCCACATAGCAGAAC-
Cluster_3.mafft_consensus       GACCGAGTTGCTCTTGCCCGGCG-TCAACACGGGATAATACCGCGCCACATAGCAGAAC-
```

```
Cluster_5.mafft_consensus       GACCGAGTTGCTCTTGCCCGGCG-TCAACACGGGATAATACCGCGCCACATAGCAGAAC-
Cluster_2.mafft_consensus       GACCGAGTTGCTCTTGCCCGGCG-TCAACACGGGATAATACCGCGCCACATAGCAGAAC-
Cluster_6.mafft_consensus       GACCGAGTTGCTCTTGCCCGGCG-TCAACACGGGATAATACCGCGCCACATAGCAGAACT
Cluster_1.mafft_consensus       GACCGAGTTGCTCTTGCCCGGCG-TCAACACGGGATAATACCGCGCCACATAGCAGAAC-
Cluster_4.mafft_consensus       GACCGAGTTGCTCTTGCCCGGCG-TCAACACGGGATAATACCGCGCCACATAGCAGAAC-
                                ********** * *  ******* ***********************************

Cluster_11.mafft_consensus      -TTTAAAAGTTC---ATCATTGGAAAACGTTCTTCGGGG--GAAACTCTC-ATGATC-TT
Cluster_13.mafft_consensus      TTTTAAAAGTGCTC-ATCATTGGAAAACGTTCTTCGGGGC-GAAACTC-CGATGATC-TT
Cluster_8.mafft_consensus       TTTTAAAAGTGCTC-ATCATTGGAAAACGTTCTTCGGGC--GAAACTCTCAAGGATC-TT
Cluster_7.mafft_consensus       TTTTAAAAGTGCTCTATCATTGGAAAACGTTCTTCGGGGC-GAAACTCTCAAGGATC-TT
Cluster_9.mafft_consensus       -TTTAAAAGTGCTC-ATCATTGGAAAACGTTCTTCGGGG--GAAAACCTCAAGGATCTTT
Cluster_3.mafft_consensus       -TTTAAAAGTGCTC-ATCATTGGAAAACGTTCTTCGGGGC-AAAACTCTCAAGGATC-TT
Cluster_5.mafft_consensus       -TTTAAAAGTGCTC-ATCATTGGAAAAGGTT-TTCGGGGCGAAAACTCTCAAGGATC-TT
Cluster_2.mafft_consensus       -TTTAAAAGTGCTC-ATCATTGGAAAACGTTCTTCGGGGC-AAAACTCTCAAGGATC-TT
Cluster_6.mafft_consensus       TTTTAAAAGTGCTC-ATCATTGGAAAACGTTCTTCGGGGCGAAAACTCTCAAGGATC-TT
Cluster_1.mafft_consensus       -TTTAAAAGTGCTC-ATCATTGGAAAACGTTCTTCGGGGCGAAAACTCTCAAGGATC-TT
Cluster_4.mafft_consensus       -TTTAAAAGTGCTC-ATCATTGGAAAACGTTCTTCGGGGCGAAAA--CTCAAGGATC-TT
                                 ********* *   ************ *** ******    ***  * * * **** **

Cluster_11.mafft_consensus      ACCGCTGTTGAGATCCAGTTCGATGTAACCCACCTGTGCACCCAACTGATTCTTCAGCAT
Cluster_13.mafft_consensus      ACCGCTGCTGAGATCCAGTTCGATGTAACCCACTCGTGCACCCAACTGATTCTTCAGCAT
Cluster_8.mafft_consensus       ACCGCTGTTGAGATCCAGTTCGATGTAACCCACTCGTGCACCCAACTGA-TCTTCAGCAT
Cluster_7.mafft_consensus       ACCGCTGTTGAGATCCAGTTCGATGTAACCCACTCGTGCACCCAACTGA-TCTTCAGCAT
Cluster_9.mafft_consensus       ACCGCTGTTGAGATCCAGTTCGATGTAACCCACTCGTGCACCCAACTGA-TCTTCAGCAT
Cluster_3.mafft_consensus       ACCGCTGTTGAGATCCAGTTCGATGTAACCCACTCGTGCACCCAACTGA-TCTTCAGCAT
Cluster_5.mafft_consensus       ACCGCTGTTGAGATCCAGTTCGATGTAACCCACTCGTGCACCCAACTGA-TCTTCAGCAT
Cluster_2.mafft_consensus       ACCGCTGTTGAGATCCAGTTCGATGTAACCCACTCGTGCACCCAACTGA-TCTTCAGCAT
Cluster_6.mafft_consensus       ACCGCTGTTGAGATCCAGTTCGATGTAACCCACTCGTGCACCCAACTGA-TCTTCAGCAT
Cluster_1.mafft_consensus       ACCGCTGTTGAGATCCAGTTCGATGTAACCCACTCGTGCACCCAACTGA-TCTTCAGCAT
Cluster_4.mafft_consensus       ACCGCTGTTGAGATCCAGTTCGATGTAACCCACTCGTGCACCCAACTGA-TCTTCAGCAT
                                ******* *************************  ************** **********

Cluster_11.mafft_consensus      C-TTTACTTTCACCAGCGTTTCTGGGTGAGCAAAAACAGGAAGGCAAAATGCCGCAAAAA
Cluster_13.mafft_consensus      CTTTTACTTTCACCAGCGTTTCTGGGTGAGCAAAAACAGGAAGGCAAAATGCCGCAAAAA
Cluster_8.mafft_consensus       CTTTTACTTTCACCAGCGTTTCTGGGTGAGC-AAAACAGGAAGGCAAAATGCCGCAAAAA
Cluster_7.mafft_consensus       C-TTTACTTTCACCAGCGTTTCTGGGTGAGC-AAAACAGGAAGGCAAAATGCCGCAAAAA
Cluster_9.mafft_consensus       CTTTTACTTTCACCAGCGTTTCTGGGTGAGC-AAAACAGGGAGGCAAAATGCCGCAAAAA
Cluster_3.mafft_consensus       CTTTTACTTTCACCAGCGTTTCTGGGTGAGCAAAAACAGGAAGGCAAAATGCCGCAAAAA
Cluster_5.mafft_consensus       CTTTTACTTTCACCAGCGTTTCTGGGTGAGCAAAAACAGGAAGGCAAAATGCCGCAAAAA
Cluster_2.mafft_consensus       CTTTTACTTTCACCAGCGTTTCTGGGTGAGC-AAAACAGGAAGGCAAAATGCCGCAAAAA
Cluster_6.mafft_consensus       CTTTTACTTTCACCAGCGTTTCTGGGTGAGCAAAAACAGGAAGGCAAAATGCCGC-AAAA
Cluster_1.mafft_consensus       CTTTTACTTTCACCAGCGTTTCTGGGTGAGC-AAAACAGGAAGGCAAAATGCCGCAAAAA
Cluster_4.mafft_consensus       CTTTTACTTTCACCAGCGTTTCTGGGTGAGC-AAAACAGGAAGGCAAAATGCCGCAAAAA
                                * ***************************** ******** ************** ****

Cluster_11.mafft_consensus      GGGAATAAGGGCGACACGGGAAATGTTGAATACTCATACTTCTTC-CTTTTCAATATTAT
Cluster_13.mafft_consensus      GGGAATAAGGGCGACAC-GGAAATGTTGAATACTCATACTCTTCCCTTTTTCAATATTA-
Cluster_8.mafft_consensus       GGGAATAAGGGCGACAC-GGAAATGTTGAATACTCATACTCTTCCTTTTTTCAATATTA-
Cluster_7.mafft_consensus       GGGAATAAGGGCGACAC-GGAAATGTTGAATACTCATACTCTTCC-TTTTTCAATATTA-
Cluster_9.mafft_consensus       GGGAATAAGGGCGACAC-GGAAATGTTGAATACTCATACTCTTCC-TTTTTCAATATTA-
Cluster_3.mafft_consensus       GGGAATAAGGGCGACAC-GGAAATGTTGAATACTCATACTCTTCC-TTTTTCAATATTA-
Cluster_5.mafft_consensus       GGGAATAAGGGCGACAC-GGAAATGTTGAATACTCATACTCTTCC-TTTTTCAATATTA-
Cluster_2.mafft_consensus       GGGAATAAGGGCGACAC-GGAAATGTTGAATACTCATACTCTTCC-TTTTTCAATATTA-
Cluster_6.mafft_consensus       GGGAATAAGGGCGACAC-GGAAATGTTGAATACTCATACTCTTCC--TTTTCAATATTA-
Cluster_1.mafft_consensus       GGGAATAAGGGCGACAC-GGAAATGTTGAATACTCATACTCTTCC-TTTTTCAATATTA-
Cluster_4.mafft_consensus       GGGAATAAGGGCGACAC-GGAAATGTTGAATACTCATACTCTTCC--TTTTCAATATTA-
                                ***************** **********************  * *  ************

Cluster_11.mafft_consensus      TGTTGAAGCATTTATCAGGGTTATTGTCTCATGAGCGGGATATGCATATTTGAATGTATT
Cluster_13.mafft_consensus      --TTGAAGCATTTATCAGGGTTATTGTCTCATGAGCGG---ATACATATTTGAATGTATT
Cluster_8.mafft_consensus       --TTGAAGCATTTATCAGGGTTATTGTCTCATGAGCGG---ATACATATTTGAATGTATT
Cluster_7.mafft_consensus       --TTGAAGCATTTATCAGGGTTATTGTCTCATGAGCGG---ATACATATTTGAATGTATT
Cluster_9.mafft_consensus       --TTGAAGCATTTATCAGGGTTATTGTCTCATGAGCGG---ATACATATTTGAATGTATT
Cluster_3.mafft_consensus       --TTGAAGCATTTATCAGGGTTATTGTCTCATGAGCGG---ATACATATTTGAATGTATT
Cluster_5.mafft_consensus       --TTGAAGCATTTATCAGGGTTATTGTCTCATGAGCGG---ATACATATTTGAATGTATT
Cluster_2.mafft_consensus       --TTGAAGCATTTATCAGGGTTATTGTCTCATGAGCGG---ATACATATTTGAATGTATT
Cluster_6.mafft_consensus       --TTGAAGCATTTATCAGGGTTATTGTCTCATGAGCGG---ATACATATTTGAATGTATT
Cluster_1.mafft_consensus       --TTGAAGCATTTATCAGGGTTATTGTCTCATGAGCGG---ATACATATTTGAATGTATT
Cluster_4.mafft_consensus       --TTGAAGCATTTATCAGGGTTATTGTCTCATGAGCGG---ATACATATTTGAATGTATT
                                  ************************************   ** ****************

Cluster_11.mafft_consensus      TAG-AAAAATAAACAAATA-GGGTTCC--GCGCACATTT-CCCCGAAAAGTGCCACCT-G
Cluster_13.mafft_consensus      TAG-AAAAATAAACAAATA-GGGTTCC-AGCGCACATTT-CCCCGAAAAGTGCCACCT-G
Cluster_8.mafft_consensus       TAG-AAAAATAAACAAATA-GGGTTCC--GCGCACATTT-CCCCGAAAAGTGCCACCTGG
Cluster_7.mafft_consensus       TAGAAAAAATAAACAAATA-GGGTTCC--GCGCACATTT-CCCCGAAAAGTGCCACCT-G
Cluster_9.mafft_consensus       TAGAAAAAATAAACAAATAGGGGTTCC--GCGCACATTT-CCCCGAAAAGTGCCACCT-G
```

```
Cluster_3.mafft_consensus       TAG-AAAAATAAACAAATAGGGGTTCC--GCGCACATTTCCCCCGAAAAGTGCCACCT-G
Cluster_5.mafft_consensus       TAG-AAAAATAAACAAATA-GGGTTCCAAGCGCACATTT-CCCCGAAAAGTGCCACCT-G
Cluster_2.mafft_consensus       TAG-AAAAATAAACAAATAGGGGTTCC--GCGCACATTT-CCCCGAAAAGTGCCACCT-G
Cluster_6.mafft_consensus       TAG-AAAAATAAACAAATA-GGGTTCC--GCGCACATTT-CCCCGAAAAGTGCCACCT-G
Cluster_1.mafft_consensus       TAG-AAAAATAAACAAATA-GGGTTCC--GCGCACATTT-CCCCGAAAAGTGCCACCT-G
Cluster_4.mafft_consensus       TAG-AAAAATAAACAAATA-GGGTTCC--GCGCACATTT-CCCCGAAAAGTGCCACCT-G
                                *** *************** *******  ********** ****************** *

Cluster_11.mafft_consensus      ACGTCTAAGAAAACCATTATTATCATGACATTAACCTAT-AAAAATAGGCGTATCACGAG
Cluster_13.mafft_consensus      ACGTCTAAG-AAACCATTATTATCATGACATTAACCTAT-AAAAATAGGCGTATCACGAG
Cluster_8.mafft_consensus       ACGTCTAAG-AAACCATTATTATCATGACATTAACCTAT-AAAAATAGGCGTATCACGAG
Cluster_7.mafft_consensus       ACGTCTAAG-AAACCATTATTATCATGACATTAACCTATAAAAAATAGGCGTATCACGAG
Cluster_9.mafft_consensus       ACGTCTAAG-AAACCATTATTATCATGACATTAACCTAT-AAAAATAGGCGTATCACGAG
Cluster_3.mafft_consensus       ACGTCTAAG-AAACCATTATTATCATGACATTAACCTAT-AAAAATAGGCGTATCACGAG
Cluster_5.mafft_consensus       ACGTCTAAG-AAACCATTATTATCATGACATTAACCTAT-AAAAATAGGCGTATCACGAG
Cluster_2.mafft_consensus       ACGTCTAAG-AAACCATTATTATCATGACATTAACCTAT-AAAAATAGGCGTATCACGAG
Cluster_6.mafft_consensus       ACGTCTAAG-AAACCATTATTATCATGACATTAACCTATAAAAAATAGGCGTATCACGAG
Cluster_1.mafft_consensus       ACGTCTAAG-AAACCATTATTATCATGACATTAACCTAT-AAAAATAGGCGTATCACGAG
Cluster_4.mafft_consensus       ACGTCTAAG-AAACCATTATTATCATGACATTAACCTAT-AAAAATAGGCGTATCACGAG
                                ********* ***************************** ********************

Cluster_11.mafft_consensus      GCCCTTTCGTCTTCAAGAATTCCTATGGAGTCAAAGATTCAAATAGAGGA-CCTAACAGA
Cluster_13.mafft_consensus      GCCCTTTCGTCTTCAAGAATTCCCATGGAGTCAAAGATTCAAATAGAGGA-CCTAACAGA
Cluster_8.mafft_consensus       GCCCTTTCGTCTTCAAGAATTCCCATGGAGTCAAAGATTCAAATAGAGGACCCTAACAGA
Cluster_7.mafft_consensus       GCCCTTTCGTCTTCAAGAATTCCCATGGAGTCAAAGATTCAAATAGAGGA-CCTAACAGA
Cluster_9.mafft_consensus       GCCCTTTCGTCTTCAAGAATTCCCATGGAGTCAAAGATTCAAATAGAGGA-CCTAACAGA
Cluster_3.mafft_consensus       GCCCTTTCGTCTTCAAGAATTCCCATGGAGTCAAAGATTCAAATAGAGGA-CCTAACAGA
Cluster_5.mafft_consensus       GCCCTTTCGTCTTCAAGAATTCCCATGGAGTCAAAGATTCAAATAGAGGA-CCTAACAGA
Cluster_2.mafft_consensus       GCCCTTTCGTCTTCAAGAATTCCCATGGAGTCAAAGATTCAAATAGAGGA-CCTAACAGA
Cluster_6.mafft_consensus       GCCCTTTCGTCTTCAAGAATTCCCATGGAGTCAAAGATTCAAATAGAGGA-CCTAACAGA
Cluster_1.mafft_consensus       GCCCTTTCGTCTTCAAGAATTCCCATGGAGTCAAAGATTCAAATAGAGGA-CCTAACAGA
Cluster_4.mafft_consensus       GCCCTTTCGTCTTCAAGAATTCCCATGGAGTCAAAGATTCAAATAGAGGA-CTTAACAGA
                                *********************** ************************** * *******

Cluster_11.mafft_consensus      ACTCGCCGTAAAGACTGGCGAACAGTTCATACAGAGTCTCTTACGACTC-AATGACAAGA
Cluster_13.mafft_consensus      ACTCGCTGCAAAGACTGGCGAACAGTTCATACAGAGTCTC-TACGACTC-AATGACAAGA
Cluster_8.mafft_consensus       ACTCGCCGTAAAGACTGGCGAACAGTTCATACAGAGTCTCTTACGACTC-AATGACAAGA
Cluster_7.mafft_consensus       ACTCGCCGTAAAGACTGGCGAACAGTTCATACAGAGT-TCTTACGACTC-AATGACAAGA
Cluster_9.mafft_consensus       ACTCGCCGTAAAGACTGGCGAACAGTTCATACAGAGTCTCTTACGACTCCAATGACAAGA
Cluster_3.mafft_consensus       ACTCGCCGTAAAGACTGGCGAACAGTTCATACAGAGTCTCTTACGACTC-AATGACAAGA
Cluster_5.mafft_consensus       ACTCGCCGTAAAGACTGGCGAACAGTTCATACAGAGTCTCTTACGACTCAAATGACAAGA
Cluster_2.mafft_consensus       ACTCGCCGTAAAGACTGGCGAACAGTTCATACAGAGTCTCTTACGACTC-AATGACAAGA
Cluster_6.mafft_consensus       ACTCGCCGTAAAGACTGGCGAACAGTTCATACAGAGTCTCTTACGACTC-AATGACAAGA
Cluster_1.mafft_consensus       ACTCGCCGTAAAGACTGGCGAACAGTTCATACAGAGTCTCTTACGACTC-AATGACAAGA
Cluster_4.mafft_consensus       ACTCGCCGTAAAGACTGGCGAACAGTTCATACAGAGTCTCTTACGACTC-AATGACAAGA
                                ****** * **************************** ** ******** **********

Cluster_11.mafft_consensus      AGAAAATCTTCGTCAACATGGTGGAGCACGACAC-CTTTTTTTCTCCCAAAATATCAAAG
Cluster_13.mafft_consensus      AGAAAATCTTCGTCAACATGGTGGAGCACGACACGCTTGTCTACTCCAAAAATATCAAAG
Cluster_8.mafft_consensus       AGAAAATCTTCGTCAACATGGTGGAGCACGACACGCTTGTCTACTCCAAAAATATCAAAG
Cluster_7.mafft_consensus       AGAAAATCTTCGTCAACATGGTGGAGCACGACACGCTTGTCTACTCCAAAAATATCAAAG
Cluster_9.mafft_consensus       AGAAAATCTTCGTCAACATGGTGGAGCACGACACGCTTGTCTACTCCAAAAATATCAAAG
Cluster_3.mafft_consensus       AGAAAATCTTCGTCAACATGGTGGAGCACGACACGCTTGTCTACTCCAAAAATATCAAAG
Cluster_5.mafft_consensus       AGAAAATCTTCGTCAACATGGTGGAGCACGACACGCTTGTCTACTCC-AAAATATCAAAG
Cluster_2.mafft_consensus       AGAAAATCTTCGTCAACATGGTGGAGCACGACACGCTTGTCTACTCCAAAAATATCAAAG
Cluster_6.mafft_consensus       AGAAAATCTTCGTCAACATGGTGGAGCACGACACGCTTGTCTACTCCAAAAATATCAAAG
Cluster_1.mafft_consensus       AGAAAATCTTCGTCAACATGGTGGAGCACGACACGCTTGTCTACTCCAAAAATATCAAAG
Cluster_4.mafft_consensus       AGAAAATCTTCGTCAACATGGTGGAGCACGACACGCTTGTCTACTCC-AAAATATCAAAG
                                ********************************** *** * * **** ************

Cluster_11.mafft_consensus      ATACAGTCTCAGAAGACCAAA-GGGCAATTGAGACTTTTCAACAAAGGGTAATATCCGAA
Cluster_13.mafft_consensus      ATACAGTCTCAGAAGACCAAAGGGGCAATTGAGACTTTTCAACAAAGGGTAATATCCGAA
Cluster_8.mafft_consensus       ATACAGTCTCAGAAGACCAAA-GGGCAATTGAGACTTTTCAACAAAGGGTAATATCCGGA
Cluster_7.mafft_consensus       ATACAGTCTCAGAAGACCAAA-GGGCAATTGAGACTTTTCAACAAAGGGTAATATCCGAA
Cluster_9.mafft_consensus       ATACAGTCTCAGAAGACCAAA-GGGCAATTGAGACTTTTCAACAAAGGGTAATATCCGAA
Cluster_3.mafft_consensus       ATACAGTCTCAGAAGACCAAA-GGGCAATTGAGACTTTTCAACAAAGGGTAATATCCGGA
Cluster_5.mafft_consensus       ATACAGTCTCAGAAGACCAAA-GGGCAATTGAGACTTTTCAACAAAGGGTAATATCCGGA
Cluster_2.mafft_consensus       ATACAGTCTCAGAAGACCAAA-GGGCAATTGAGACTTTTCAACAAAGGGTAATATCCGAA
Cluster_6.mafft_consensus       ATACAGTCTCAGAAGACCAAA-GGGCAATTGAGACTTTTCAACAAAGGGTAATATCCGAA
Cluster_1.mafft_consensus       ATACAGTCTCAGAAGACCAAA-GGGCAATTGAGACTTTTCAACAAAGGGTAATATCCGAA
Cluster_4.mafft_consensus       ATACAGTCTCAGAAGACCAAA-GGGCAATTGAGACTTTTCAACAAAGGGTAATATCCGAA
                                ********************* ************************************ *

Cluster_11.mafft_consensus      A-CCTCCTCGGATTCCATTGCCCAGCTATCTGTCACTTTATTGTGAAGATAGTGGAAAGG
Cluster_13.mafft_consensus      AACCTCCTCGGATTCCATTGCCCAGCTATCTGTCACTTTATTGTGAAGATAGTGGAAAAG
Cluster_8.mafft_consensus       AACCTCCTCG-ATTCCATTGCCCAGCTATCTGTCACTTTATTGTGAAGATAGTGGAAAAG
Cluster_7.mafft_consensus       A-CCTCCTCGGATTCCATTGCCCAGCCATCTGTCACTTTATTGTGAAGATAGTGGAAAA-
```

```
Cluster_9.mafft_consensus      ACCTTCCTCGGATTCCATTGCCCAGCTATCTGTCACTTTATTGTGAAGATAGTGGAAAAG
Cluster_3.mafft_consensus      AACCTCCTCGAATTCCATTGCCCAGCCATCTGTCACTTTATTGTGAAGATAGTGGAAAAG
Cluster_5.mafft_consensus      AACCTCCTCGGATTCCATTGCCCAGCTATCTGTCACTTTATTGTGAAGATAGTGGAAAAG
Cluster_2.mafft_consensus      A-CCTCCTCGGATTCCATTGCCCAGCCATCTGTCACTTTATTGTGAAGATAGTGGAAAAG
Cluster_6.mafft_consensus      A-CCTCCTCGGATTCCATTGCCCAGCCATCTGTCACTTTATTGTGAAGATAGTGGAAAAG
Cluster_1.mafft_consensus      AACCTCCTCGGATTCCATTGCCCAGCTATCTGTCACTTTATTGTGAAGATAGTGGAAAAG
Cluster_4.mafft_consensus      ACCCTCCTCGGATTCCATTGCCCAGCTATCTGTCACTTTATTGTGAAGATAGTGGAAAAG
                               * * ****** *************** *******************************

Cluster_11.mafft_consensus     GAAGGTGGCTCCTACAAATGCCATCATT-GCGATAAAGGAAAGGCCATCGTTGAAGATGC
Cluster_13.mafft_consensus     GAAGGTGGCTCCTACAAATGCCATCATT-GCGATAAAGGAAAGGCCATCGTTGAAGATGC
Cluster_8.mafft_consensus      GAAGGTGGCTCCTACAAATGCCATCATTGGCGATAAAGGAAAGGCTATCGTTGAAGATGC
Cluster_7.mafft_consensus      GAAGGTGGCTCCTACAAATGCCATCATT-GCGATAAAGGAAAGGCCATCGTTGAAGATGC
Cluster_9.mafft_consensus      GAAGGTGGCTCCTACAAATGCCATCATT-GCGATAAAGGAAAGGCCATCGTTGAAGATGC
Cluster_3.mafft_consensus      GAAGGTGGCTCCTACAAATGCCATCATT-GCGATAAAGGAAAGGCCATCGTTGAAGATGC
Cluster_5.mafft_consensus      GAAGGTGGCTCCTACAAATGCCATCATT-GCGATAAAGGAAAGGCCATCGTTGAAGATGC
Cluster_2.mafft_consensus      GAAGGTGGCTCCTACAAATGCCATCATT-GCGATAAAGGAAAGGCCATCGTTGAAGATGC
Cluster_6.mafft_consensus      GAAGGTGGCTCCTACAAATGCCATCATT-GCGATAAAGGAAAGGCCATCGTTGAAGATGC
Cluster_1.mafft_consensus      GAAGGTGGCTCCTACAAATGCCATCATT-GCGATAAAGGAAAGGCCATCGTTGAAGATGC
Cluster_4.mafft_consensus      GAAGGTGGCTCCTACAAATGCCATCATT-GCGATAAAGGAAAGGCCATCGTTGAAGATGC
                               **************************** **************** **************

Cluster_11.mafft_consensus     -TCCTGCCGACAGTGGTCCCAAAGATGGGA-CCCCACCCACGAGGAGCATCGTGG-AAAA
Cluster_13.mafft_consensus     TCTCTGCCGACAGTGGTCCCAAAGATGGA--CCCCACCCACGAGGAGCATCGTG--AAAA
Cluster_8.mafft_consensus      TTTCTGCCGACAGTGGTCCCAAAGATGGAC-CCCCACCCACGAGGAGCATCGTGG-AAAA
Cluster_7.mafft_consensus      TCTCTGCCGACAGTGGTCCCAAAGATGGAC-CCCCACCCACGAGGAGCATCGTGG-AAAA
Cluster_9.mafft_consensus      TCTCTGCCGACAGTGGTCCCAAAGATGGACTCCCCACCCACGAGGAGCATCGTGG-AAAA
Cluster_3.mafft_consensus      CTTCTGCCGACAGTGGTCCCAAAGATGGAC-CCCCACCCACGAGGAGCATCGTGG-AAAA
Cluster_5.mafft_consensus      TTTCTGCCGACAGTGGTCCCAAAGATGGAC-CCCCACCCACGAGGAGCATCGTGG-AAAA
Cluster_2.mafft_consensus      -CTTTGCCGACAGTGGTCCCAAAGATGGAC-CCCCACCCACGAGGAGCATCGTGGGAAAA
Cluster_6.mafft_consensus      -TTCTGCCGACAGTGGTCCCAAAGATGGAC-CCCCACCCACGAGGAGCATCGTGGAAAAA
Cluster_1.mafft_consensus      -CTCTGCCGACAGTGGTCCCAAAGATGGAC-CCCCACCCACGAGGAGCATCGTGG-AAAA
Cluster_4.mafft_consensus      -CTCTGCCGACAGTGGTCCCAAAGATGGAC-CCCCACCCACGAGGAGCATCGTGGAAAAA
                                   ************************   ***********************  ****

Cluster_11.mafft_consensus     GAAGACGTTCCAACCACGTCTTCAAAGCAAGTGGATTGATGTGATATCTCCCGACTGACG
Cluster_13.mafft_consensus     GAAGACGTTCCAACCACGTCTTCAAAGCAAGTGGATTGATGTGATATCTCC--ACTGAC-
Cluster_8.mafft_consensus      GAAGACGTTCCAACCACGTCTTCAAAGCAAGTGGATTGATGTGATATCTCC--ACTGAC-
Cluster_7.mafft_consensus      GAAGACGTTCCAACCACGTCTTCAAAGCAAGTGGATTGATGTGATATCTCC--ACTGAC-
Cluster_9.mafft_consensus      GAAGACGTTCCAACCACGTCTTCAAAGCAAGTGGATTGATGTGATATCTCC--ACTGAC-
Cluster_3.mafft_consensus      GAAGACGTTCCAACCACGTCTTCAAAGCAAGTGGATTGATGTGATATCTCC--ACTGAC-
Cluster_5.mafft_consensus      GAAGACGTTCCAACCACGTCTTCAAAGCAAGTGGATTGATGTGATATCTCC--ACTGAC-
Cluster_2.mafft_consensus      GAAGACGTTCCAACCACGTCTTCAAAGCAAGTGGATTGATGTGATATCTCC--ACTGAC-
Cluster_6.mafft_consensus      GAAGACGTTCCAACCACGTCTTCAAAGCAAGTGGATTGATGTGATATCTCC--ACTGAC-
Cluster_1.mafft_consensus      GAAGACGTTCCAACCACGTCTTCAAAGCAAGTGGATTGATGTGATATCTCC--ACTGAC-
Cluster_4.mafft_consensus      GAAGACGTTCCAACCACGTCTTCAAAGCAAGTGGATTGATGTGATATCTCC--ACTGAC-
                               ***************************************************  ******
```

The large discrepancy in sequence between the clusters is due to the relatively high error rate of MinION sequencing.

Sanger confirmed sequence

5’GCGGTAAGATCCTTGAGAGTTTTCGCCCCGAAGAACGTTTTCCAATGATGAGCACTTTTAAAGTTCTGCTATGTGGCGCGGTATTATCCCGT
GTTGACGCCGGGCAAGAGCAACTCGGTCGCCGCATACACTATTCTCAGAATGACTTGGTTGAGTACTCACCAGTCACAGAAAAGCATCTTACGG
ATGGCATGACAGTAAGAGAATTATGCAGTGCTGCCATAACCATGAGTGATAACACTGCGGCCAACTTACTTCTG|CATAACCAATTAGTGGCAA
GTTGGATTCACTTGAATAAAACAAACCTTTGTCGATTGTTCCTCGGAGGTATCTGAATAAATGTTTGATACCATTCCAGTGTCTTTGTGTCGGG
GAAGAACTAAATCTTGCTAATAAGTTTACAGAAAAAGCAATGTCTGGTCGG 3’

| transition site

Amplicon sequence, including event-specific primers and probe

G1

5’- GGATGGCATGACAGTAAGAGAATTATGCAGTGCTGCCATAACCATGAGTGATAACACTGCGGCCAACTTACTTCTG|CATAACCAATTAGTGGC - 3’
3’- CCTACCGTACTGTCATTCTCTTAATACGTCACGACGGTATTGGTACTCACTATTGTGACGCCGGTTGAATGAAGAC|GTATTGGTTAATCACCG - 5’

G2

Clusters based on MinION data

```
Cluster_167.mafft_consensus      TGCTGCAACTCT----CAGGGCCAGGCGGTGAAGGGCAATCAGCTGTTGCCCGTCTCACT
Cluster_158.mafft_consensus      TGCTGCAACTCTCTC-CAGGGCCAGGCGGTGAAGGGCAATCAGCTGTTGCCCGTCTCACT
Cluster_116.mafft_consensus      TGCTGCAACTCTCTCCCAGGGCCAGGCGGTGAAGGGCAATCAGCTGTTGCCCGTCTCACT
Cluster_15.mafft_consensus       TGCTGCAACTCTCT--CAGGGCCAGGCGGTGAAGGGCAATCAGCTGTTGCCCGTCTCACT
Cluster_65.mafft_consensus       TGCTGCAACTCTCT--CAGGGCCAGGCGGTGAAGGGCAATCAGCTGTTGCCCGTCTCACT
Cluster_68.mafft_consensus       TGCTGCAACTCTCT--CAGGGCCAGGCGGTGAAGGGCAATCAGCTGTTGCCCGTCTCACT
Cluster_106.mafft_consensus      TGCTGCAACTCTCT--CAGGGCCAGGCGGTGAAGGGCAATCAGCTGTTGCCCGTCTCACT
Cluster_187.mafft_consensus      TGCTGCAACTCTCTCAAAGGGCCAGGCGGTGAAGGGCAATCAGCTGTTGCCCGTCTCACT
                                 ************     *******************************************


Cluster_167.mafft_consensus      -GTGAAAAG-AAAAACCACCCCAGTACATT-AAAACGTCCGCAATGTGTACAAGATTGTG
Cluster_158.mafft_consensus      GGTGAAAAG-AAAAACCACCCCAGTACATT-AAAACGTCCGCAATGTGTACAAGATTGTG
Cluster_116.mafft_consensus      GGTGAAAAG-AAAAACCACCCCAGTACATTAAAAACGTCCGCAATGTGTACAAGATTGTG
Cluster_15.mafft_consensus       GGTGAAAAA-AAAAAACCCCCAGTACATT-AAAACGTCCGCAATGTGTACAAGATTGTG
Cluster_65.mafft_consensus       GGTGAAAAG-AAAAACCACCCCAGTACATT-AAAACGTCCGCAATGTGTACAAGATTGTG
Cluster_68.mafft_consensus       GGTGAAAAG-AAAAACCACCCCAGTACATT-AAAACGTCCGCAATGTGTACAAGATTGTG
Cluster_106.mafft_consensus      GGTGAAAAG--AAAACCACCCCAGTACATT-AAAACGTCCGCAATGTGT----TATTAAG
Cluster_187.mafft_consensus      GGTGAAAAGAAAAAACCACCCCAGTACATTAAAAACGTCCGCAATGTGT----TATTAAG
                                  *******   **** * ************ ******************     ***  *


Cluster_167.mafft_consensus      GTGCTTCAA-CAGCATGTGCAATTTGCCAGTGCTCAAACTTAATACCGTGTAGATTATAC
Cluster_158.mafft_consensus      GTGCTTCAACCAGCATGTGCAATTTGCCAGTGCTCAAACTTAATACCGTGTAGATT-TAC
Cluster_116.mafft_consensus      GTGCTTCAA-CAACATGTGCAATTTGCCAGTGCTCAAACTTAATACCGTGTAGATTATAC
Cluster_15.mafft_consensus       GTGCTTCAA-CAGCATGTGCAATTTGCCAGTGCTCAAACTTAATACCGTGTAGATTATAC
Cluster_65.mafft_consensus       GTGCTTCAA-CAGCATGTGCAATTTGCCAGTGCTCAAACTTAATACCGTGTAGATTATAC
Cluster_68.mafft_consensus       GTGCTTCAA-CAGCATGTGCAATTTGCCAGTGCTCAAACTTAATACCGTGTAGATTATAC
Cluster_106.mafft_consensus      TTGTCT----AAGCGT---CAATTTGTTTACACCACAATCTTGTACA--ACAAATTG---
Cluster_187.mafft_consensus      TTGTCCTAA---GCGT---CAATTTGTTTACACCACAATCTTGTACA--ACAAATTG---
                                  **          * *   *******      *   **  *  ***     * ***


Cluster_167.mafft_consensus      CGTGTAGATA-CACGGTATAA--TTTTCGGCG-AGGGCGGTCATCCT--CAAAGGTGGCT
Cluster_158.mafft_consensus      CGTGTAGATA-CACGGTATAAATTTTTCGGCGAAGGGCAGTCATCCT---CAAGGTGGCT
Cluster_116.mafft_consensus      CGTGTAGATA-CACGGTATAA-TTTTTCGGCGAAGGGCGGTCATCCTC-CCAAGGTGGCT
Cluster_15.mafft_consensus       CGTGTAGATA-CACGGTATAA-TTTTTCGGCGAAGGGCGGTCATCCT--CCAAGGTGGCT
Cluster_65.mafft_consensus       CGTGTAGATA-CACGGTATAATTTTTTCGGCGAAGGGCGGTCATCCT--CCAAGGTGGCT
Cluster_68.mafft_consensus       CGTGTAGATA-CACGGTATAA-TTTTTCGGCGAAGGGCGGTCATCCT--CCAAGGTGGCT
Cluster_106.mafft_consensus      -ACGTATCTACCACAGTATAATTTTTTCGGCGAAGGGCGGTCATCCTCATCAATTTGTTT
Cluster_187.mafft_consensus      -ACGTATCTA---CAGTATAA--TTTTCGGCGAAAGGCGGTCATCCTCATCAATTTGTTT
                                    ***  **   * ******  ********* * *** ********    **  **  *


Cluster_167.mafft_consensus      ACGCCGACACGCTT--AGTGCCGATCCAGCACAACTTGATCCCTTTTAGCTGCTTCCCTA
Cluster_158.mafft_consensus      ACGCCGACACCTTTGAATTGCCGATCCAGCACAAC--GAT-CCTTTTAGCTGCTTCCCTA
Cluster_116.mafft_consensus      ACGCCGACACCTTTGAATTGCCGATCCAGCACAACTTGAT-CCCTTTAGCTGCTTCCCTA
Cluster_15.mafft_consensus       ACGCCGACACCTTTGAATTGCCGATCCAGCACAACTTGAT-CCTTTTAGCTGCTTCCCTA
Cluster_65.mafft_consensus       ACGCCGACACCTTTGAATTGCCGATCCAGCACAACTTGATCCCTTTTAGCTGCTTCCCTA
Cluster_68.mafft_consensus       ACGCCGACACCTTTGAATTGCCGATCCAGCACAACTTGATCCCTTTTAGCTGCTT-CCTA
Cluster_106.mafft_consensus      ACACCAC---------ATTGCGGA-----CGAAAAATTATACCGTGTAGATACGTCCGCA
Cluster_187.mafft_consensus      ACACCC----------ATTGCGGA-----CGAAA----ATTCCGTGTAGATACGT-----
                                 ** **           * *** **     *  **    ** ** * *** * * *

Cluster_167.mafft_consensus      TCGGTGAGATTATGTTGGTCG---ATGCTGAATTT---AGC----GACCATTGTA-TTTA
Cluster_158.mafft_consensus      TCGGTGAGATTATGTTGGTCG---ATGCTGGATTT---AGC----GACCATTGTA-TTTA
Cluster_116.mafft_consensus      TCGGTGAGATTATGTTGGTCG---ATGCTGAATTT---AGC----GACCATTGTATTTTA
Cluster_15.mafft_consensus       TCGGTGAGATTATGTTGGTCG---ATGCTGAATTT---AGC----GACCATTGTA-TTTA
Cluster_65.mafft_consensus       TCGGTGAGATTATGTTGGTCG---ATGCTGAATTT---AGC----GACCATTGTA-TTTA
Cluster_68.mafft_consensus       TCGGTGAGATTATGTTGGTCG---ATGCTGAATTT---AGC----GACCATTGTA-TTTA
Cluster_106.mafft_consensus      AT-GTGTTATTAAGTTGTCTAAGCATGTGCAATTTGCCAGTGCTCAAACAGGTTAGGATA
Cluster_187.mafft_consensus      ---GCAATGTGTTATTAGTTG--TCTGGCATATTT---GCCGTGCTCCAAAGGTAGGATA
                                    *     *    **         **    ****              *   **   **

Cluster_167.mafft_consensus      TTCATTTGATCCTGCGAGTCAAAAGTTTGAAACATTTGTGGTCGCCAAATTCCCAATATC
Cluster_158.mafft_consensus      TTCATTTGATCCTGCGGGTCAAA-GTTTGAAACATTTGTGGTCGCCAAATTCCCAATT--
Cluster_116.mafft_consensus      TTCATTTGATCCTGCGAGTCAAAAGTTTGAAACATTTGTGGTCGCCAAATT-CTAATAT-
Cluster_15.mafft_consensus       TTCATTTGATCCTGCGAGTCAAAAGTTTGAAACATTTGTGGTCGCCAAATT--CAATAT-
Cluster_65.mafft_consensus       TTCATTTGATCCTGCGAGTCAAAAGTTTGAAACATTTGTGGTCGCCAAATTCCCAATATC
Cluster_68.mafft_consensus       TTCATTTGATCCTGCGAGTCAAAAGTTTGAAACATTTGTGGTCGCCAAATTCCCAATATC
Cluster_106.mafft_consensus      TTCCTCAGGATC----AGCCAAAGCTTCTGCTGCTTTCTGGTTAAC-------CTACATC
Cluster_187.mafft_consensus      TTCCTCAGGATC----AGCCAAAGCTTCTGCTGCTTTCTGGTTACCCA------------
```

```
                                 *** *  *   *     * ****  **       *** ****   *

Cluster_167.mafft_consensus      CAAAATGGACACCTCTACCCGCATATC--TTTTTTCAACCAAATTTTCAATCCGCTCAAT
Cluster_158.mafft_consensus      CAAAATGGACACCTCTA-CCGCATATC--TTTTTTC------------------------
Cluster_116.mafft_consensus      CAAAATGGACACCTCTACCCGCATATCTTTTTTTTCAACCAAA-TTTCAATCCGCTCAAT
Cluster_15.mafft_consensus       CAAAATGGACACCTCTA-CCGCATATC--TTTTTCA---------TTCAATC--CTCAAT
Cluster_65.mafft_consensus       CAAAATGGACACCTCTACCCGCATATC--TTTTTTCAACCAAA-TTTCAATCCGCTCAAT
Cluster_68.mafft_consensus       CAAAATGGACACCTCTACCCGCATATC--TTTTTTCAACCAAA-TTTCAATCCGCTCAAT
Cluster_106.mafft_consensus      ATACATGGCGAAATC-ACTCAGAAAAC--TAGGTTCTAATAA---TGAAAAAGGATTCGC
Cluster_187.mafft_consensus      TCACATGGCGAAATC-ACTCAGAAAAC--TAGTTCTAATAA----TGAAAAAGGATTCGC
                                   * ****  *  ** *  *  * * *  *   *
```

The large discrepancy in sequence between the clusters is due to the relatively high error rate of MinION sequencing.

Sanger confirmed sequence

5’TGCTGCAACTCTCTCAGGGCCAGGCGGTGAAGGGCAATCAGCTGTTGCCCGTCTCACTGGTGAAAAGAAAAACCACCCCAGTACATTAAAAA
CGTCCGCAATGTGTACAAGATTGTGGTGCTTCAACAGCATGTGCAATTTGCCAGTGCTCAAACTTAATACCGTGTAGATTATACCGTGTAGATA
CACGGTATAATTTTTCGGCGAAGGGCGGTCATCCTCCAAGGTGGCTACGCCG|ACACCTTTGAATTGCCGATCCAGCACAACTTGATCCCTTTT
AGCTGCTTCCCTATCGGTGAGATTATGTTGGTCGATGCTGAATTTAGCGACCATTGTATTTATTCATTTGATCCTGCGAGTCAAAAGTTTGAAA
CATTTGTGGTCGCCAAATTCCCAATATCC 3’

| transition site

Amplicon sequence, including event-specific primers and probe

G2

5’- GCCAGTGCTCAAACTTAATACCGTGTAGATTATACCGTGTAGATACACGGTATAATTTTTCGGCGAAGGGCGGTCATCCTCCAAGGTGGCTACGCCG|ACACCTTTGAATTGCCGATCCAG - 3’
3’- CGGTCACGAGTTTGAATTATGGCACATCTAATATGGCACATCTATGTGCCATATTAAAAAGCCGCTTCCCGCCAGTAGGAGGTTCCACCGATGCGGC|TGTGGAAACTTAACGGCTAGGTC - 5’
